# Quality-by-design based RP-HPLC analytical method for the estimation of quercetin in silver-metal organic frameworks (AgMOF): a Box-Behnken optimization approach

**DOI:** 10.1038/s41598-025-32452-6

**Published:** 2025-12-18

**Authors:** Anoushka Mukharya, Srinivas Mutalik

**Affiliations:** Department of Pharmaceutics, Manipal College of Pharmaceutical Sciences, Manipal Academy of Higher Education, Manipal, 576104 Karnataka India

**Keywords:** Quercetin, Box-Behnken design, Metal organic frameworks, Diabetic wounds, Design of experiments (DoE), Quality by design (QbD), Chemistry, Environmental sciences

## Abstract

An efficient, accurate, sustainable, and reproducible chromatographic method for Quercetin estimation was developed, optimized, and further validated using a Design of Experiments approach in compliance with ICH guidelines. The Box-Behnken Quality-by-Design optimized critical parameters, yielding robust performance, excellent linearity (R^2^ = 0.9996, 0.05–50 μg/mL), and high sensitivity (LOD: 40.27 ng/mL, LOQ: 122.03 ng/mL). Deliberate modifications evaluated by analysis of variance verified the method’s significance (P < 0.0001). The method utilises a Phenomenex HyperClone C18 LC Column (250 × 4.6 mm; 5 μm, 130 Å) with an isocratic mobile phase consisting of acidified water (0.1% v/v Triethylamine, pH 3.0 ± 0.05) and Acetonitrile (55:45% v/v). Forced degradation studies revealed that quercetin was stable under mild acidic, photolytic, and oxidative conditions at room temperature but suffered significant degradation under basic, strong acidic, and overall accelerated conditions (60 °C for 6 h), indicating sensitivity to harsh environments. Greenness evaluation results show an AGREE score of 0.6 and a favorable ComplexGAPI profile, highlighting minimal energy consumption during the 12-min run and moderate solvent/reagent use. The method’s efficiency and repeatability promote robust quercetin quantification, with wide applications in various pharmaceuticals and nanomaterials, as demonstrated by its effective use in silver-based Metal–Organic Frameworks in this study.

**Supplementary Information:**

The online version contains supplementary material available at 10.1038/s41598-025-32452-6.

## Introduction

Quercetin (QCT), a naturally formed flavonoid, has already been scientifically established to hold significant anti-inflammatory, antioxidant, antibacterial, and wound-healing activities. It is one of the most extensively researched bioflavonoids to alleviate the symptoms of metabolic diseases. By influencing macrophage polarization, lowering inflammatory cytokine production while boosting tissue regeneration, QCT also facilitates diabetic wound healing, as demonstrated by existing research^1^. Among diabetic animal models, topical QCT stimulates collagen deposition, fibroblast activity, wound contraction, and antioxidant enzyme function, all of which contributes to a more rapid and effective wound healing^2^. Nevertheless, QCT’s reduced skin permeability, instability, and insufficient solubility affect its therapeutic adaptation. Facilitating localized and sustained release of QCT at the wounded area, technologically advanced drug delivery nanoparticulate systems like Metal-Organic Frameworks (AgMOFs) present a promising approach to navigate around these restrictions. This is expected to enhance QCT’s overall bioavailability and therapeutic effectiveness in diabetic wound healing, while also reducing systemic side effects.

Diabetic foot ulcers (DFUs) and other diabetic wounds are non-healing wounds that remain a major clinical challenge owing to delayed wound healing attributed to long-term inflammation, peripheral neuropathy, oxidative distress, deficient angiogenesis, or diminished microcirculation, the entirety of which is made more acute in diabetic patients^3^. The primary objective of the standard of treatment for DFUs is to mitigate physical strain, deal with the biochemical deficiencies that underlie diabetic wound repair, including reactive oxygen species, prolonged inflammation, and reduced angiogenesis^4^. A promising addition to wound therapy and topical QCT administration is a comprehensive nanoparticulate system (AgMOFs) that deals with a multifaceted approach^5^. AgMOFs are capable of carrying therapeutic substances precisely to the wound bed by relatively painless penetration of the stratum corneum without breaching the epidermal barrier^6^. Due to its antifungal and antioxidant properties, QCT is a promising candidate for faster and more efficient diabetic wound healing. According to the latest research, silver-based compounds may serve as less invasive, nontoxic, biodegradable, and low-immunogenic carriers for administering active substances like QCT. Moreover, AgMOFs can be combined with additional components that promote healing or can be ornamented to achieve optimal healing outcomes^7^.

In essence, QCT’s therapeutic applications go substantially beyond simply mending wounds. With the objective to address a range of medical ailments, including cancer, diabetes, cardiovascular disease, neurodegenerative conditions, allergic reactions, stomach ulcers, osteoporosis, arthritis, and multiple inflammatory and pathogenic conditions, QCT has been widely researched and developed for topical, oral, transdermal, intravenous and intranasal administration, as presented in Table 1. This versatile nature has triggered an ample amount of research into novel delivery methods that enhance the drug’s solubility, stability, and bioavailability, ranging from tablets, gels, emulsions, microneedle patches and even nanoparticulate systems^8^.

**Table 1:** Quercetin’s Therapeutic Versatility: Combining Route of Administration, Therapeutic Dose, Formulation Platforms, and Therapeutic Targets.

Route	Dose	Formulation Types	Medical Conditions	References
Oral	500–1000 mg/day (FDA Generally Regarded as Safe approved for 500 mg/day—GRN no. 341)	Tablets, capsules, powders, phytosomes, nanoemulsions, Suspensions	Cardiovascular disease, cancer (colon, prostate, leukemia), Neurodegenerative conditions, diabetes, allergy, asthma, peptic ulcers, osteoporosis, arthritis, inflammatory bowel disease	^9–14^
Topical	0.1–2% w/w (typical gel/cream concentration)	Ethosomal gels, hydrogels, microemulsions, SLN, NLC, nanovesicles, creams, proniosomes	Wound healing (including diabetic wounds), UV-induced skin damage, anti-ageing, inflammation, Diabetic foot ulcers, psoriasis, eczema, keratoconus, ocular surface disorders	^15–19^
Transdermal	Typically, 10–100 mg/patch (no standardized or FDA-approved transdermal therapeutic dose)	Patches, films, nanocarrier-loaded films	Systemic delivery for chronic inflammation, metabolic syndrome, cardiovascular disease, and cancer therapy	^20–22^
Intravenous	About 100–400 mg single IV dose	Solutions, nanoformulations, prodrugs (e.g., QC12)	Oncology (prostate, leukemia), acute therapy, clinical trials for advanced cancer	^23–26^
Intranasal	0.02–10 mg/kg (rat models)	Sprays, nanotransferosomal inhalation gels	Neuroprotection, Anxiety, CNS delivery (experimental for Parkinson’s and Alzheimer’s disease)	^27–30^

Although QCT has numerous pharmacologically beneficial characteristics, pharmacokinetic research, formulation development, and quality control are all dependent upon the precise estimation of QCT in several different and most often sophisticated delivery systems. Among the various spectroscopic methods used to quantify a drug, the High-performance liquid chromatography (HPLC) technique remains the most accurate and convenient for sensitive and specific detection of QCT in different formulations across a diverse therapeutic dose^31^. For HPLC method development, the "one-factor-at-a-time" (OFAT) approach, which modifies one independent component at a time until the optimal solution is found, provides only a broad understanding of the procedure’s potential and repeatability. Hence, the OFAT technique may not suggest the most appropriate methodology. The Quality-by-Design (QbD) approach, on the other hand, provides a systematic screening process for the establishment of analytical methods. The application of Analytical QbD principles improves and reinforces analytical method development by making it easier to identify the root sources of variation in a scientific and risk-based manner. Furthermore, a method’s capacity to signal stability is compromised when degraded peaks form under severe conditions or after extended storage, placing the selectivity observed on a non-degraded sample at risk. To address these issues, stress-induced forced degradation studies have been conducted under specific stress conditions^32^.

The ultimate purpose of this research is to establish and further validate a consistent and robust Reverse Phase High Performance Liquid Chromatography (RP-HPLC) analytical method for quantifying QCT in various innovative formulations, such as AgMOFs loaded with QCT, intended to promote the healing of diabetic wounds. This work attempts to quantify QCT via a robust HPLC method, enhancing its therapeutic application in various formulations. This leads the way in helping improve prospects in diabetic, non-healing wound therapy alongside various chronic disease conditions by combining forefront analytical and drug delivery advances in technology.

## Materials

Quercetin (QCT) was procured from Sigma Aldrich, Bangalore, Karnataka, India (purity, ≥ 95% HPLC grade)**.** Methanol (MeOH), Acetonitrile (ACN) of HPLC grade (purity: 99.8%) and Hydrochloric acid (HCl, 35% pure Analytical Reagent) were acquired from FINAR Limited (Ahmedabad, Gujarat, India). Pellets of sodium hydroxide (NaOH, purity ≥98%) and Hydrogen Peroxide (H_2_O_2_, 30%) were obtained from Himedia Labs Pvt. Ltd. (Mumbai, India). Triethylamine (TEA, purity 99%) was acquired from Loba Chemie Pvt. Ltd., Mumbai, India. Ortho-phosphoric acid (OPA, 88%) was brought via Merck Ltd, Mumbai, India. A nylon membrane filter of 0.22 µm was purchased from Riviera Glass Pvt. Ltd (Mumbai, India). All additional materials used were of analytical grade, unless otherwise specified.

## Methodology

### Instrumentation and apparatus

A HPLC system (Shimadzu, Kyoto, Japan) with a CBM-20A Prominence system controller, LC-10 AD pump, DGU-20A5 degasser unit, column oven, SIL-10AXL auto-sampler, and sample cooling unit was employed to perform the chromatographic separation of QCT. The results were gathered using UV-Vis lamp with a multi-wavelength detector (PDA-M20A) prominence for the chromatographic investigations. LabSolutions version 5.57, the system control software, was utilized for data collection, output chromatogram monitoring, and peak integration. HyperClone BDS C18, LC Column (250 x 4.6 mm, 130 Å, 5 µm) was acquired from Phenomenex (Hyderabad, India) for separation and analysis of QCT. Design Expert®, version 9.0.1.0 (Stat-Ease Inc., Minneapolis, USA), was implemented for trials involving the design of experiments (DoE), response modelling, and desirability calculation. Standard materials were weighed using a Saffron SES204 analytical balance (Saffron Electronics scale, Surat, India). A bath sonicator (GT Sonic, Guangdong GT Ultrasonic Co. Ltd, China) was utilized to degas the mobile phase solution after it had been filtered through a membrane filter of 0.22-micron specification (Riviera Glass Pvt. Ltd., Mumbai, India) combined with a vacuum-filtration system (Tarsons Products Pvt. Ltd., Kolkata, India). The mobile phase pH was adjusted utilizing a digital pH meter (Systronics India Ltd., Model 335, Ahmedabad, Gujarat, India). Ultrapure water (Type 1 MilliQ) was taken from a Millipore Direct Q® 3 UV-purification system (Millipore Corporation, USA) to prepare the mobile phase and samples.

### Preparation of mobile phase and the standard solutions

**Mobile phase:** HPLC-grade Acetonitrile (ACN) was used as the organic phase (Channel A). The aqueous phase (Channel B) consists of Milli-Q water with TEA (0.1% v/v), further acidified with OPA by adjusting the pH to 3.

**Standard solutions and Sample dilutions of Quercetin:** By precisely weighing and vortexing 10 mg of QCT in 5 mL of methanol (MeOH; diluent) for 4 to 5 minutes in a 10 mL volumetric flask, the primary standard solution of QCT (Stock solution) was prepared. After adding diluent and bringing the volume up to 10 mL, the final solution was bath-sonicated for two minutes to reach a final 1 mg/mL concentration. The 1 mg/mL primary stock solution was again diluted to obtain a 100 µg/mL solution, which was further used to prepare different samples in a predefined linear concentration. Linearity samples with strengths varying from 0.05 to 50 µg/mL were made by properly diluting the standard QCT stock solutions with the diluent. For forced degradation assessments, a 1 mg/mL standard solution of QCT was utilized. For HPLC analysis, it was serially diluted to make a 1 µg/mL solution in MeOH.

### Development of HPLC method and optimization

#### Selection of wavelength

Theoretically, there are two main absorption bands in the UV-visible range of 240 to 400 nm based on the two cyclic rings in the QCT molecule. A working standard of 10 µg/mL was obtained via further diluting the standard solution of 1 mg/mL in MeOH for the purpose of establishing the wavelength required for QCT estimation. The working standard solution was then analyzed in the 200 to 400 nm range utilizing a UV–Vis double-beam spectrophotometer (UV-1601PC, Shimadzu, Japan) to find absorption maxima (λmax) using MeOH as a blank.

### Chromatographic conditions

QCT was effectively separated by RP-HPLC system utilizing a Phenomenex HyperClone C-18 column (BDS 130 Å, 5 μm, 250 mm × 4.6 mm). The most effective separation of QCT was achieved by incorporating a mobile phase consisting of 0.1% v/v TEA in Type 1 water (pH was adjusted to 3.0±0.05) and ACN (55:45% v/v) via an isocratic elution after the influence of various mobile phase compositions were assessed. The pH of the buffer (aqueous phase) was modified using OPA solution (80% v/v). Before analysis, the mobile phase was subjected to a 20-minute bath sonication after being filtered through a 0.45 μm membrane attached to a vacuum filtration setup. The mobile phase was kept at a constant 0.95 mL/min flow rate. Each sample went through a total run time of 12 minutes and 20 μL volume of injection. The C18 column was kept at 25°C, while the QCT detection wavelength was measured at 371 nm. The autosampler was set at 4 °C by default.

### DoE-based RP-HPLC method optimization

#### Evaluation of risk assessment

To sequentially determine the most Critical Method Parameters (CMPs) that can influence the separation efficiency of the developed isocratic RP-HPLC method for quantifying QCT, the Ishikawa (fishbone) diagram was established. By graphically representing the possible factors that might influence Critical Analytical Attributes (CAAs), including peak area, number of theoretical plates (NTP), retention time (Rt) (min), tailing factor (Tf), peak symmetry, and sensitivity, the diagram served as a risk assessment tool. A targeted risk-based approach was used to identify essential components as determined by literature review, evaluation, and pre-existing research without conducting an initial screening study.

#### Identification of critical variables (independent and dependent) and optimization of method by Box-Behnken design (BBD)

The impact of several process variables, including the ratio of mobile phase or percentage of organic phase (ACN), column temperature, and flow rate, on results, including peak Tf, NTP, peak area and Rt, was scrutinized using research evaluation and preliminary method development trials. Additionally, a working range was defined for every single variable after preliminary research incorporating a One Factor at a Time (OFAT) approach to screen among those process parameters/variables and pinpoint ones that had a substantial impact on the method’s responses.

The HPLC technique was further refined and optimized using BBD with three Critical Method Parameters (factors): organic phase (A), Column Oven Temperature (B), and flow rate (C), each having two levels, Low (-1) and High (+1) values set. BBD was chosen over Central Composite Design (CCD) because it demanded a reduced number of runs to evaluate the three factors. The main effects, factor interactions, and equations of independent factors over dependent responses were analysed utilizing BBD from Design Expert® software (Version 13.0.5.0, Stat-Ease Inc., Minneapolis, MN, USA) by means of the polynomial equation, and the factors were further statistically enhanced. Table S1 (Supplementary Information) data summarises the dependent variables or the Responses (CAA) and the independent variables or the Factors (CMP) along with their code, units and levels predetermined based on the preliminary HPLC data. Parameters for subsequent method optimization were chosen based on the desirability determined by the BBD prediction.

### Method validation

The US Pharmacopoeia, International Conference on Harmonization’s (ICH) Q2 (R1) guidelines were implemented in the overall validation of the RP-HPLC analytical method that was designed for the estimation of QCT. The implemented method was validated for specificity, linearity, system suitability, precision, sensitivity, robustness, accuracy and degradation studies^33–35^**.**

#### System suitability

To evaluate the system suitability parameter, a QCT analyte strength of 1 μg/mL solution was injected 6 times (n=6) to test the suitability/compatibility of the instruments, columns and analysts, to determine the stability and repeatability of the HPLC output for the desired analytical method. The peak area, Tf, Rt, and NTP, were established^36^. In compliance with the USP ICH recommendations, the system must comply with the following requirements for suitability, Tf ≤ 2, theoretical plates must have a column efficiency of ≥ 2000, and the RSD value must be less than 1% for n ≥ 5^37,38^.

#### Specificity

The contribution of the blank’s interference was assessed by injecting a standard QCT solution of 1 μg/mL sample, including the blank diluent, to ascertain the specificity of the devised analytical procedure^39^. Any interferences at the QCT retention times were further assessed^40^.

#### Linearity (calibration curve)

Linearity is the potential of a test to yield results that are directly proportionate to the analyte’s concentration. The concentration range of 0.05 to 50 μg/mL was used to determine the linearity of QCT. After the QCT primary stock solution (1,000 μg/mL) was adequately diluted, concentrations of 0.05, 0.1, 0.25, 0.5, 0.75, 1.0, 5.0, 10.0, and 50.0 μg/mL were obtained. The linearity and range of QCT were assessed by analyzing six injections for each concentration prepared. The graph between peak area (y-axis) and concentration (x-axis) was utilized to determine the linear regression and correlation coefficient.

#### Sensitivity

The limit of detection (LOD) represents a minimal amount of analyte in a test sample that is detectable yet is not quantifiable to a precise value. The limit of quantitation (LOQ) is, by definition, the minimum quantity of analyte within a test sample that can be numerically quantified with desirable accuracy and precision. LOD and LOQ were determined using Equations (1) and (2) below.

1 $$\text{LOD }= \frac{3.3*\sigma }{Slope}$$

2 $$\text{LOQ }= \frac{10*\sigma }{Slope}$$

The slope obtained from the calibration curve equation and the standard deviation 'σ' of the linear regression line’s y-intercept were used to calculate the sensitivity of the analytical approach^34,41^.

#### Robustness

The robustness of the method was assessed by tweaking the parameters of the implemented method one at a time. An analytical method’s potential to tolerate minor but deliberate changes to method parameters is a sign of its resilience. Quantitative, qualitative, and mixture-related factors are all included in the robustness test. Qualitative aspects, including solvents, different chromatographic columns, and chemical origins, were examined throughout the initial method development stage of the current examination of the suggested RP-HPLC method. Hence, these qualitative factors were not considered for the robustness test due to their minimal impact on the analytical method. The following quantitative variables were used to estimate the robustness of the proposed analytical method: (i) Analyzing at 373 and 369 nm, two distinct wavelengths, (ii) injection volume (20±2 μL), (iii) flow rate of the mobile phase (0.95±0.10 mL/min), (iv) organic phase used (45±2% v/v), (v) adjusting the column temperature to 23℃ and 27℃, and (vi) pH of the mobile phase (3.0±0.2). For each condition, responses, including theoretical plates, peak area, and Tf, were examined in three repeated injections, and the percentage RSD was calculated.

#### Precision

The ICH Guidelines state that a method’s precision is evaluated by examining its capacity to produce reproducible results under similar experimental conditions. Six replicates of three distinct concentration levels, namely, low quality control at 750 ng/mL, medium quality control at 1250 ng/mL, and high-quality control at 2500 ng/mL, were injected into the RP-HPLC to evaluate the intra-day and inter-day precision (also known as a repeatability study) of the implemented method. While the inter-day precision was conducted on different consecutive days, the intraday precision was carried out in the morning and evening of the same day. After noting the peak area, the percentage relative standard deviation (% RSD) was computed.

#### Accuracy

The accuracy of an analytical procedure is the degree of agreement between the value observed and the value considered to be a known setpoint or a conventional true value. The accuracy of the proposed method was evaluated through recovery analysis, which involved spiking a predetermined quantity of QCT at three different levels. The three distinct concentrations of QCT dilutions (0.75, 1.25, and 2.5 μg/mL) were obtained by properly diluting the stock solution of QCT. These concentrations were then injected into HPLC (n=3). The percentage recovery and percentage RSD were calculated for every concentration according to the following equations (3) and (4), respectively^42^.

3 $${\% Actual Recovery}=\frac{Actual concentration recovered}{Theoretical concentration} x 100$$

4 $${\% RSD }(\text{Relative Standard Deviation})=\frac{Standard Deviation of Peak Area}{Average Peak Area} x 100$$

#### 24 hours Bench-top stability

To ascertain the bench-top stability of QCT in a standard concentration solution of 1µg/mL, the precision analysis samples were kept at room temperature. The bench-top test samples and freshly produced samples using MeOH as diluent were injected into the chromatographic system four times after a 24-hour period. Utilizing the subsequent equation (5), the similarity index was determined ^43,44^.

5 $$\text{Similarity Index }= \frac{\text{Mean Peak Area of old standard }\times \text{ Concentration of new standard}}{\text{Mean Peak Area of new standard }\times \text{ Concentration of old standard}}$$

In the above formula, the concentration in the old and fresh samples is determined from the calibration curve, respectively. The mean peak area of the old/standing standard is the Bench-top QCT samples after 24 hours of being kept at ambient temperature, and the mean peak area of the new/fresh standard is that of the freshly prepared QCT samples.

#### Stress-induced degradation studies

To evaluate the stress-testing capability of the implemented RP-HPLC method for QCT, forced degradation studies were conducted in accordance with ICH Q1A(R2) guidelines. The aim was to assess the method’s ability to detect the drug in the presence of its degradation products under a range of stress conditions that mimic long-term and harsh storage environments.

A standard solution (1mL of 1 µg/mL concentration) of QCT was subjected to various stress settings to determine the degradation response of the devised analytical method. The first stressor was acid hydrolysis carried out at two different concentrations (0.1N HCl, mild acid and 1N HCl, strong acid), which involved combining QCT solution with 2 mL of hydrochloric acid (HCl). The second stressor was base-induced degradation, in which 0.1N (mild base) and 1N (strong base) sodium hydroxide (NaOH) was added to the QCT solution. The third stressor was oxidative hydrolysis, wherein the QCT solution was combined with 30% hydrogen peroxide (H₂O₂). Both ambient and accelerated settings were employed to execute the process of degradation. The standard drug solution and stressor blends were refluxed for 12 hrs at bench-top conditions during the course of the ambient degradation studies. For the accelerated studies, the blends were refluxed for 6 hours at 60 °C under accelerated conditions. Thermal stress was studied under dry heat conditions (80 °C for 12 hours in the solid state) and moist heat (reflux with water at 60 °C for 6 hours), while photolytic degradation was carried out by exposure to UV light. Samples were then neutralized after completing their respective exposure time in the degradation experiments, and were appropriately diluted using MeOH as the diluent for further HPLC analysis^40^.

### Developed and validated RP-HPLC method application

Utilizing the previously demonstrated, RP-HPLC validated method, the QCT loaded in the silver-based Metal Organic Framework (AgMOF) system was estimated. The implementation of the validated RP-HPLC employed method was verified by estimating the QCT entrapment efficiency and percentage of drug loading using the designed nanoparticulate formulation.

#### Formulation of QCT-loaded AgMOFs

The scalable antibacterial silver MOF carrier system was synthesized via a precipitation-based self-assembly process. Terephthalic acid and TEA were first dissolved in N,N-dimethylformamide (DMF) under continuous stirring at 500 rpm. Silver nitrate was separately dissolved in DMF and then added dropwise to the reaction mixture, allowing silver ions to coordinate with the deprotonated linkers and form Ag-MOFs, which precipitated immediately as a white solid. The resulting Ag-MOFs were collected by filtration, washed twice each with DMF and MeOH, and dried at 80 °C for 2 hours^45^. Furthermore, for the preparation of QCT-loaded silver-based MOF (QCT-AgMOFs), 30 mg of QCT and 30 mg of the prepared AgMOFs were combined with 30 mL of a water-MeOH mixture (1:1, v/v) and stirred at 500 rpm for 24 hours to facilitate the encapsulation of QCT into the AgMOF carrier system^46,47^.

#### Characterization of AgMOFs

To evaluate the AgMOFs, the overall particle size of the carrier, along with its polydispersity index (PDI) and Zeta Potential, was analyzed using the Malvern Zetasizer NANO-ZS (ZEN-3600, Malvern Analytical Limited, United Kingdom)^48^.

Furthermore, to analyze the surface morphology and structural framework of the developed silver-based nanoparticulate system (AgMOFs), Scanning electron microscopy (SEM) was used and the sample was viewed at a working distance of 8 mm. The dried AgMOFs prepared were crushed and adhered to double-sided carbon tape with aluminium stubs on one side, and thereafter sputter-coated with a very light and uniform coating of gold for better sample conductivity and imaging quality.

The drug loading (DL%) and entrapment efficiency (EE%) of the QCT-loaded silver-based metal organic frameworks (QCT-AgMOFs) were assessed by drawing 2 mL of the direct loading mixture containing 30 mg of QCT, 30 mg AgMOFs (carrier) and 30 mL of a water-MeOH mixture (1:1) after 24 hours of continuous stirring at 500 rpm. The 2 mL solution was then centrifuged at 15,000 rpm and 25 ℃. The pellet formed was basically the drug-loaded AgMOFs, while the supernatant consists of the free or unentrapped drug that was collected and kept aside for further analysis. To the remaining pellet, 1 mL of MeOH was added, the pellet was redispersed, and the sample was again subjected to centrifugation at the same configuration. This step is also known as the washing step, where the supernatant was again collected and kept for further analysis. This step was repeated about three to four times till there was only a minimal quantity of free drug analyzed from within the supernatant using the developed HPLC method. The samples were then subjected to analysis via the RP-HPLC system utilizing the above-validated analytical method. A blank solution (without drug and only MeOH) was injected into the HPLC before analyzing the unentrapped drug supernatant samples to verify peak specificity and look for any excipient interference at the Rt of QCT. Based on the calibration curve generated from QCT standards, the unknown QCT concentration was measured in the supernatant samples, and further, the value was extrapolated to the total volume of the sample in which the drug was dissolved. The EE% and the DL% of the QCT-AgMOFs were calculated based on the following equations (6) and (7), respectively^49^.

6 $$\text{EE\% }= \frac{\text{Amount of drug entrapped}}{\text{Total amount of drug added}} \times 100$$

7 $$\text{DL\% }= \frac{\text{Actual drug recovered in AgMOFs}}{\text{Total weight of formulation }(\mathrm{carrier}+\mathrm{drug})}\hspace{0.17em}\times \hspace{0.17em}100$$

### Greenness assessment of the developed QCT analytical method

The objective of green analytical chemistry (GAC) is to evaluate the impact on the environment, safety of operators, and health factors in compliance with the 12 principles of GAC. Numerous green assessment tools that offer quantitative scores indicating the environmental sustainability of analytical techniques have been developed as a result of the growing curiosity in GAC over the years. The AGREE (Analytical GREEnness Metric Approach and Software) tool was used in this study for the analysis of the implemented RP-HPLC method’s sustainability or greenness to quantify QCT^50^. With a score ranging from 0 to 1, this tool compares the method to the 12 fundamentals of GAC, where higher scores denote a more environmentally considerate and compatible solution^51^. Reagent toxicity, waste production, energy use, sample throughput, miniaturization, automated processes, and the safety of operators were among the factors that were evaluated. The rigorous AGREE assessment supported the compatibility of the implemented analytical method with GAC principles by providing an unbiased, upfront quantification of its sustainability and environmental effects.

The Complex Green Analytical Procedure Index (GAPI) assessment of the established RP-HPLC method was conducted to further examine its environmental impact and safety profile^52^. Complex GAPI produces a pentagram-shaped visual tool, where each segment represents a critical phase in the analytical workflow, including sample preparation, reagent utilization, and instrumentation^53^. Every portion that makes up the pentagram is colour-coded, where green stipulates low effect, yellow denotes medium impact, and red implies severe environmental threat, thereby offering a clear qualitative assessment of the method’s sustainability at each stage^54^.

## Results

### Development of RP-HPLC analytical method

#### Selection of wavelength

**Fig. 1:**
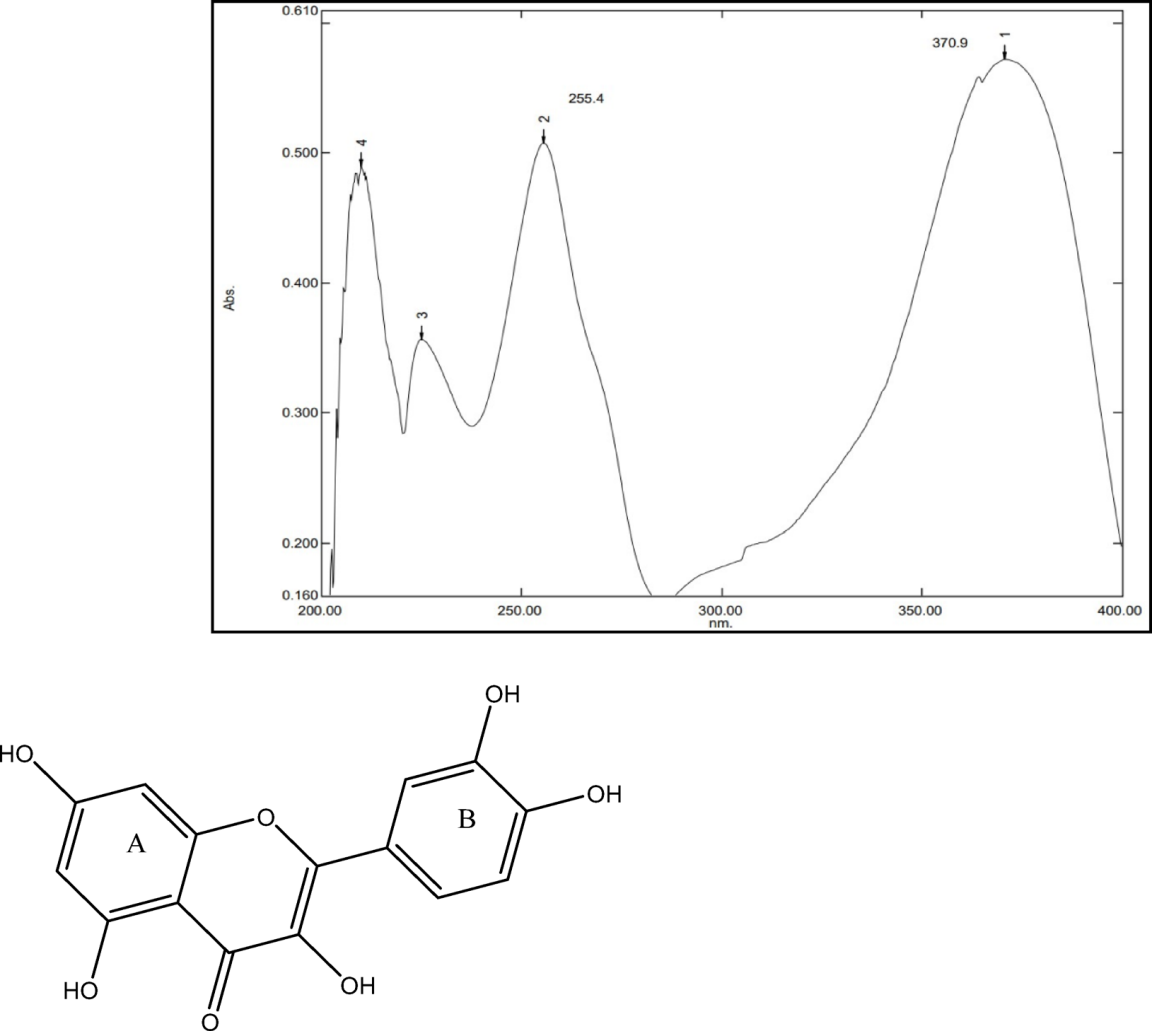
UV–visible spectrum of pure drug QCT (λ _max_ at 371 nm) and its chemical structure showcasing the two major rings detected on the UV spectrum (PubChem CID:5,280,343).

Comparing the UV spectrum in Fig. 1 with the chemical structure of QCT, Peak 1 at 370.9 nm represents ring B, which is usually within the range of 300-400 nm; whereas Peak 2 at 255.4 nm represents ring A, which is usually in the range of 240 to 300 nm^55^. Here, λ_max_ of pure drug QCT was found to be at 370.99 nm.

Further method development was carried out using the obtained λ _max_ at 371 nm wavelength for the analytical detection of QCT.

### RP-HPLC analytical method development for quercetin utilizing DOE

#### Evaluation of risk assessment

Acetonitrile was ultimately used as the organic solvent of choice during method development because it had a higher elution power in comparison to MeOH, resulting in shorter run time and sharper peaks. An isocratic elution approach was employed to preserve simplicity and repeatability in the mobile phase components, considering the method was designed for the assessment of only one analyte, QCT. To ascertain analyte stability and ideal ionization, the influence of buffer selection and pH was examined^56^. To increase technique sensitivity, the wavelength for the detection of QCT was selected in accordance with the established UV absorption maxima of QCT. To find the right equilibrium between analysis time and chromatographic resolution, flow rate evaluation was also carried out.

**Fig. 2:**
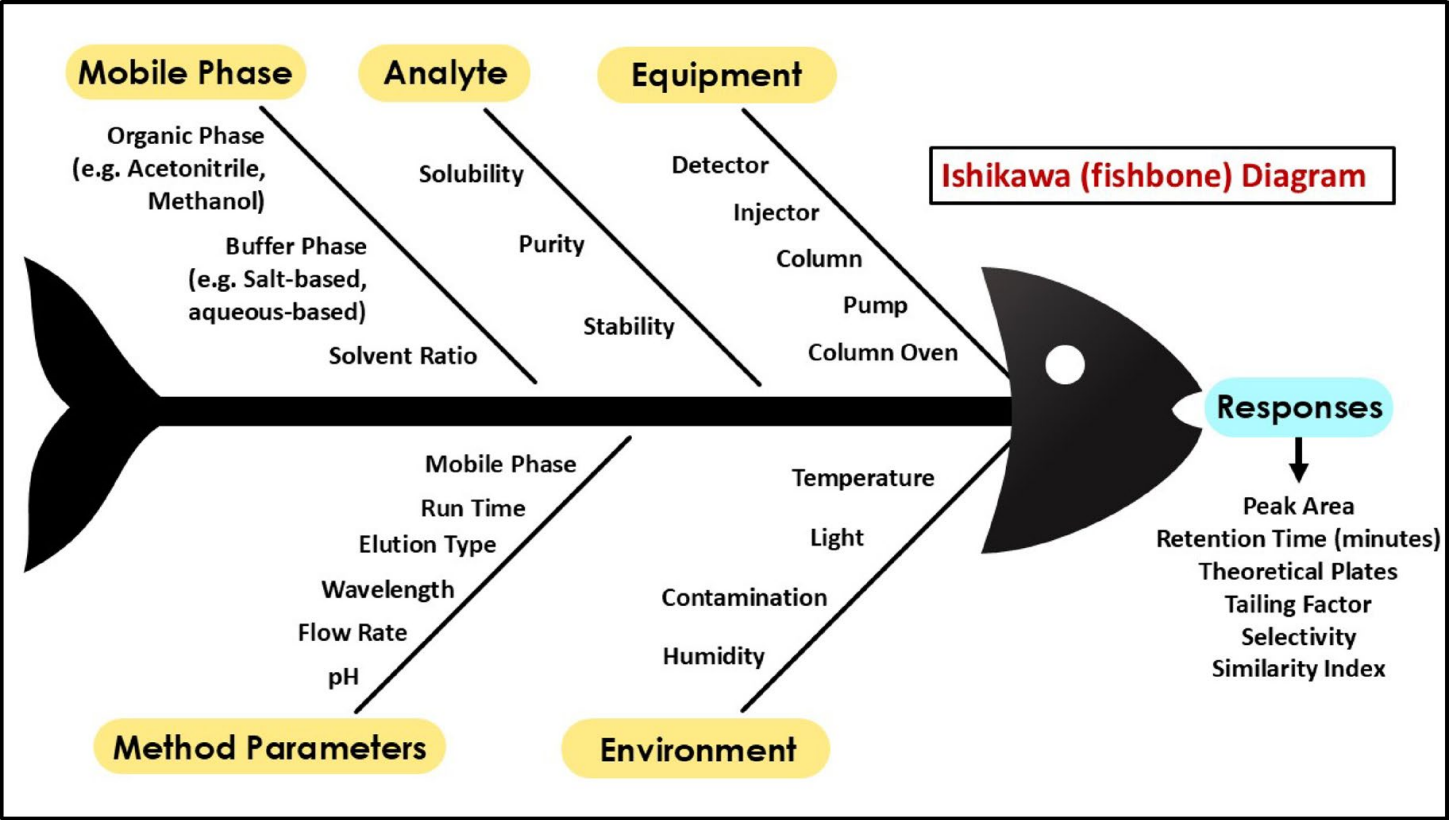
Ishikawa diagram depicting critical process variables that may alter the separation behaviour of the isocratic RP-HPLC method for QCT quantification.

Stemming from the evaluation of the risk assessment, the CMPs identified for further optimization using BBD were flow rate, ACN concentration of mobile phase and column oven temperature. An Ishikawa (Fishbone) diagram (Fig. 2) is a visual aid for systematically identifying and mapping the likely causes of a certain outcome, such as the key variables impacting the HPLC method performance. The illustration represents major groups comprising mobile phase, analyte, equipment, method parameters, and environment as primary “bones” branching from the central spine, with each reflecting potential sources of variation that might affect responses such as peak area, Rt, etc. It makes it easier to identify and select only the characteristics that have a substantial impact while leaving the others constant. The other variables in the presented Ishikawa diagram have been kept constant at their standard levels due to the fact that they have no significant impact on the overall method performance. This systematic strategy provides focused optimization by addressing the most essential components.

Effective method performance optimization was made possible by this multivariate technique, which eliminated the necessity for a preliminary screening design.

#### BBD method optimization (DoE approach)

The response surface randomized design matrix contained 17 experimental runs, of which n=5 were replicates of the centre point to approximate any experimental ambiguity or uncertainty. These Runs were then conducted on the RP-HPLC system (Shimadzu, Japan) along with the Phenomenex HyperClone BDS C18, 5 µm, 250 x 4.6 mm, 130 Å, LC Column to report the responses corresponding to the associated run. Table 2 below shows the Experimental runs carried out for the randomized Box-Behnken Design of the Experiment.

These responses were further analyzed using a statistical approach via ANOVA, specifically for a quadratic and reduced cubic models, to see if the model is significant or not and whether the parameters fall within the acceptable limits. It was found that the p-values were below 0.0500, which indicates that the model terms are significant. Even the overall F-value this large has only a 0.01% chance of occurrence due to noise, hence it implies that the models are significant in nature. In the case of the quadratic models for Peak Area (R1) and Retention time (R2), the Residual Analysis resulted in Lack of Fit F-values of 0.65 and 3.14, demonstrating their non-significant nature relative to the pure error. This means that there is a 62.34% and 14.90% chance that a Lack of Fit F-value this large could occur due to noise, which further supports the conclusion that the model is a good fit for the responses R1 and R2, respectively. Furthermore, Lack of fit cannot be evaluated statistically in reduced cubic models for Theoretical Plates (R3) and Tailing factor (R4), since all possible degrees of freedom are employed to estimate the model for responses, leaving no remaining degrees of freedom for a lack-of-fit test. Finally, the R^2^ values for all the responses are closest to 1.0 and show high significance, as shown in Table 3 below.

**Table 2:** RP-HPLC experimental data for the Box-Behnken Design.

Runs	Factor A: ACN (%)	Factor B: temperature (℃)	Factor C: flow rate (mL/min)	Response 1: peak area	Response 2: retention time (Rt) (mins)	Response 3: theoretical plates (NTP)	Response 4: tailing factor (Tf)
1	47.5	25	0.8	623,035	4.517	7977	1.127
2	47.5	35	0.8	616,564	4.348	7810.67	1.095
3	45	30	0.8	622,400	4.69	8391.67	1.157
4	45	35	0.9	548,118	4.083	7716	1.151
5	47.5	30	0.9	559,953	3.946	7293.33	1.108
6	47.5	30	0.9	557,936	3.95	7309.33	1.108
7	50	35	0.9	554,617	3.76	3841.33	0.739
8	47.5	30	0.9	565,084	3.951	7291.67	1.111
9	45	30	1	507,567	3.759	7283.33	1.162
10	50	30	0.8	622,810	4.285	3748.67	0.722
11	47.5	25	1	504,809	3.611	6854	1.119
12	47.5	30	0.9	553,191	3.951	7274.33	1.11
13	45	25	0.9	558,685	4.256	7902	1.164
14	47.5	30	0.9	554,105	3.951	7273	1.109
15	50	25	0.9	563,302	3.887	3212.67	0.776
16	50	30	1	501,233	3.45933	3547.67	0.81
17	47.5	35	1	500,162	3.48733	6649.33	1.057

**Table 3:** RP-HPLC ANOVA statistical analysis for the experimental data of the Box-Behnken design of Experiment.

Responses	Peak area (R1) (Quadratic Model)		Retention time (Rt) (R2) (quadratic model)		Theoretical plates (NTP) (R3) (reduced cubic model)		Peak tailing factor (Tf) (R4) (reduced cubic model)	
**F-value**	158.64		26060.22		17950.72		35232.32	
**p-value**	**R1 Model**	< 0.0001	**R2 Model**	< 0.0001	**R3 Model**	< 0.0001	**R4 Model**	< 0.0001
**A-ACN**	**A**	0.6909	**A**	< 0.0001	**A**	< 0.0001	**A**	< 0.0001
**B-Temperature**	**B**	0.0458	**B**	< 0.0001	**B**	0.0003	**B**	< 0.0001
**C-Flow Rate**	**C**	< 0.0001	**C**	< 0.0001	**C**	< 0.0001	**C**	< 0.0001
**A-B-C Interactions**	**AB**	0.8378	**AB**	< 0.0001	**AB**	< 0.0001	**AB**	0.0002
	**AC**	0.4712	**AC**	< 0.0001	**AC**	< 0.0001	**AC**	< 0.0001
	**BC**	0.8427	**BC**	< 0.0001	**BC**	0.2722	**BC**	0.0001
	**A²**	0.9134	**A²**	< 0.0001	**A²**	< 0.0001	**A²**	< 0.0001
	**B²**	0.3593	**B²**	0.0051	**B²**	0.0513	**B²**	0.0001
	**C²**	0.0466	**C²**	< 0.0001	**C²**	0.0017	**C²**	0.0061
**Residual Analysis**	**Lack of Fit (not significant)**		**Lack of Fit (not significant)**		**A²B**	< 0.0001	**A²B**	< 0.0001
	**F-value**	0.6496	**F-value**	3.14	**A²C**	< 0.0001	**A²C**	< 0.0001
	**p-value**	0.6234	**p-value**	0.1490	**AB²**	0.0122	**AB²**	0.0074
**R²**	0.9951		1.0000		1.0000		1.0000	
**Adjusted R²**	0.9888		0.9999		0.9999		1.0000	

Further, when the Experimental data were graphically analysed using perturbation plots and 3D Surface Model Graphs for the ANOVA Statistical Analysis results of the BBD optimization, the effect of Independent Variables (Factors) on the analysed Responses was closely observed to predict the overall trend in the factors with respect to their responses.

The final quadratic equation in terms of coded factors on the analyzed Peak Area (Response 1) was found to be as follows:

$$\begin{aligned} Peak \, Area \, = & \; + 5.581 \times 10^{5} + 649.00*A - 3796.25*B - 58879.75*C \\ & \; + 470.50*AB - 1686.00*AC + 456.00*BC + 243.35*A^{2} \\ & \; - 2116.65*B^{2} + 5205.35*C^{2} \\ \end{aligned}$$

Peak Area = + 5.581×10^5^ + 649.00*A - 3796.25*B - 58879.75*C + 470.50*AB - 1686.00*AC + 456.00*BC + 243.35*A^2^ - 2116.65*B^2^ + 5205.35*C^2^

The effect of stated factors on the analyzed Peak Area (R1) according to the Equation, Perturbation plot and 3D surface plots showed that the Flow Rate (Factor C) has significant effect compared to the Column oven Temperature (Factor B) and the amount of Organic solvent (ACN) used in the Mobile phase (Factor A) which was found to have slight effect on the Peak Area as shown in Fig. S1 in Supplementary Information.

The final quadratic equation in terms of coded factors on the analyzed Retention Time (Response 2) was found to be as follows:

$$\begin{aligned} Rt \, = & + \, 3.95 - 0.1745*A - 0.0741*B - 0.4405*C \, + \, 0.0114*AB + 0.0264*AC \\ & \; + \, 0.0113*BC \, + \, 0.0519*A^{2} - 0.0055*B^{2} + \, 0.0465*C^{2} \\ \end{aligned}$$

The effect of stated factors on the analyzed Rt (R2) according to the Equation, Perturbation plot and 3D surface plots showed that the Flow Rate (Factor C) has maximum significant effect, then comes the Organic Phase (Factor A) with medium effect and finally the Column oven Temperature (Factor B) which was found to have slight effect over the peak Rt as shown in Fig. S2 of the Supplementary Information.

The final reduced cubic equation in terms of coded factors on the analyzed Theoretical Plates (NTP) (Response 3) was found to be as follows:

$$\begin{aligned} NTP = & + 7288.333 - 2094.66*A - 92.75*B - 571.08*C \, + \, 203.66*AB \\ & \; + \, 226.84*AC - 9.59*BC - 1600.12*A^{2} - 20.21*B^{2} + \, 54.63*C^{2} \\ & \; + \, 203.41*A^{2} B \, + \, 243.75*A^{2} C - 46.33*AB^{2} \\ \end{aligned}$$

The effect of stated factors on the analysed NTP (Response 3- R3) according to the Equation, Perturbation plot and 3D surface plots showed that the Organic Phase (Factor A) has a maximum significant effect then comes the Flow Rate (Factor C) with medium effect and finally the Column oven Temperature (Factor B) which was observed to have a slight effect on the Theoretical Plates as shown in Fig. S3 of the Supplementary Information.

The final reduced cubic equation in terms of coded factors on the analyzed Peak Tailing Factor (Response 4) was found to be as follows:

$$\begin{aligned} Peak \, Tf = & + \, 1.11 - 0.1966*A - 0.0237*B - 0.0113*C - 0.0061*AB \\ & \; + 0.0206*AC - 0.0077*BC - 0.1441*A^{2} - 0.0074*B^{2} - 0.0025*C^{2} \\ & \; + 0.0111*A^{2} B \, + \, 0.0344*A^{2} C{-}0.0035*AB^{2} \\ \end{aligned}$$

The effect of stated factors over the analyzed Peak Tf (Response 4 – R4) according to the Equation, Perturbation plot, and 3D surface plots showed that the Organic Phase (Factor A) has a maximum significant effect, then comes the Column oven Temperature (Factor B) with a slight effect, and finally the Flow Rate (Factor C) which was found to have the least effect on the Peak Tf (R4) as shown in Fig. S4 (Supplementary Information).

**Fig. 3:**
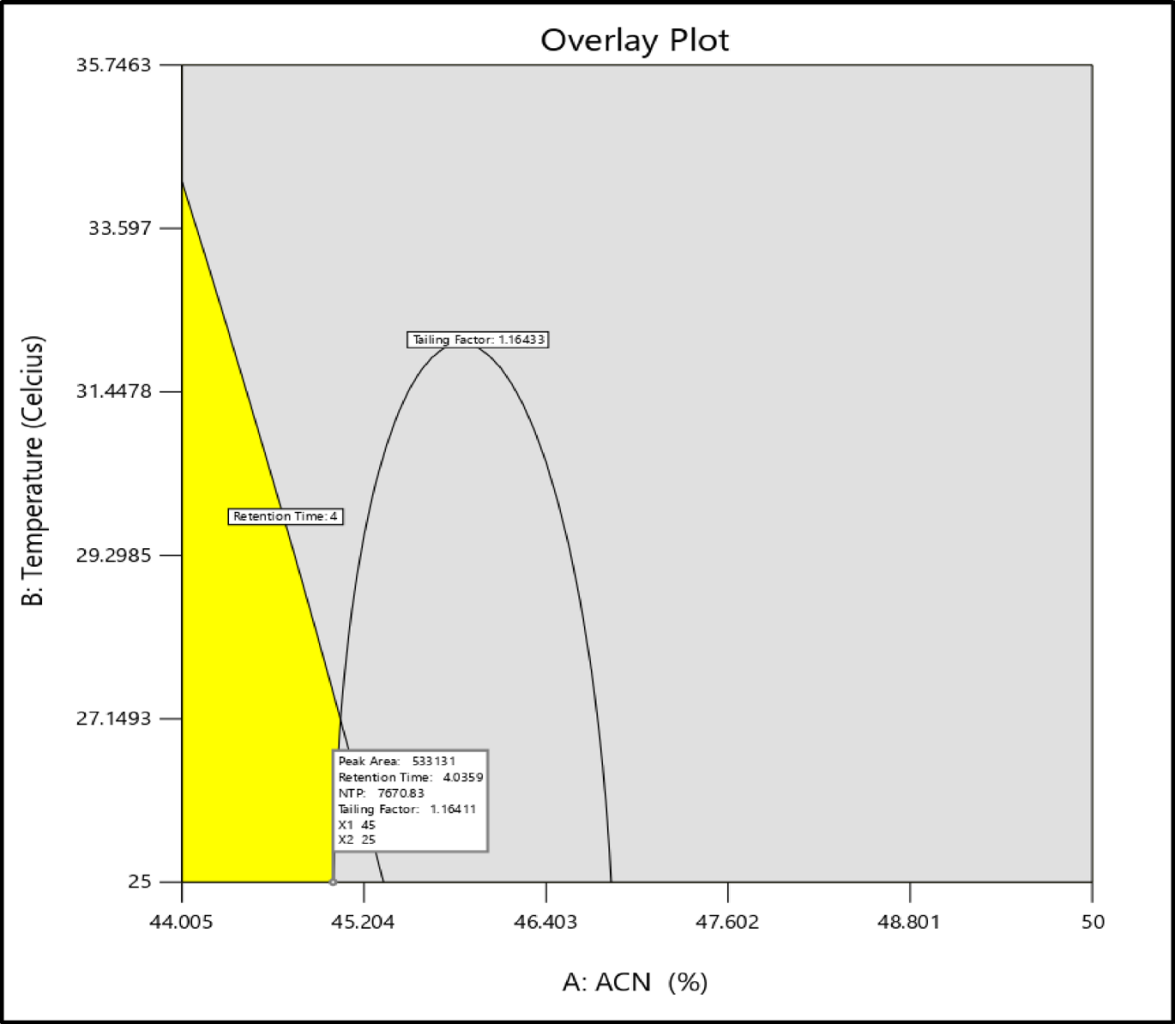
Overlay plot of Analytical RP-HPLC method-optimized Solution 1 with respect to AB interaction using Box-Behnken design of Experiment.

To eventually determine the ideal factor settings for several responses at once, the overlay plot visually identifies the “sweet spot” or optimal zone where several response criteria can be satisfied, emphasizing factor configurations that are acceptable and undesirable or the regions where predicted solutions fall within acceptable limits, as seen in Fig. 3.

The runs emphasized in this region were experimentally carried out, and the responses were further observed to see if they meet the expected parameters. The final optimized solution, most likely having a desirability of 1 or more than 0.8, corresponding to maximum reproducibility, is then selected. This solution is then confirmed and is further used to validate and assess the system’s suitability for the final HPLC analytical method.

The final selected Analytical method Parameter was that of Run 7 (Solution 1) with a desirability of 1. As clearly observed in Table S2 and Fig. S5 (Supplementary Information), Run 7 (Solution 1) has the most optimum peak, with all responses falling under acceptable ranges. This was further confirmed statistically via the Confirmation Analysis in the Design Expert® Software, as shown in Fig. S6 (Supplementary Information). The Solution was performed 6 times to assess its repeatability and statistical significance. It was found that the predicted data mean was well within the desired range with a 95% confidence level.

“Method validation” is the process of identifying a selected method’s fundamental elements through laboratory testing to ensure it can fulfil the requirements of its intended usage. In keeping with the ICH-Q2 (R1) guidelines, the optimized liquid chromatographic technique, as represented in Table S3 (Supplementary Information), was validated for several specifications, including linearity, system suitability, LOD, LOQ, robustness, precision (intra- and inter-day), and accuracy of QCT.

### System suitability

The effectiveness and steady functioning of the column, Rt, peak area, height, NTP, HETP and Tf were evaluated using six replicate injections of the standard solution containing QCT (1 μg/mL). The % RSD acceptance requirements for the Critical Analytical Attributes (CAA) were limited to a value of less than 2%. The final results observed in Fig. S6 were tabulated in Table S4 of the Supplementary Information, which were well within the acceptable limit.

### Specificity

Specificity of the developed QCT method is well supported by the PDA (photodiode array) peak purity analysis. The computed peak purity index was 0.999980 (Fig. S7 of Supplementary Information), which is significantly high and close to 100% peak purity. Furthermore, the PDA detector validated the analyte’s spectral homogeneity and optimum detection wavelength, strengthening the established method’s specificity (Fig. S1 of Supplementary Information).

### Linearity (calibration curve)

Linearity of the method was determined by examining QCT standard solutions made with serial dilutions from the working standard solution 10 times for each concentration in the 50–50000 ng/mL range. Nominal concentrations (x-axis) were plotted against the average peak area (y-axis) to estimate the specimen data.

**Fig. 4:**
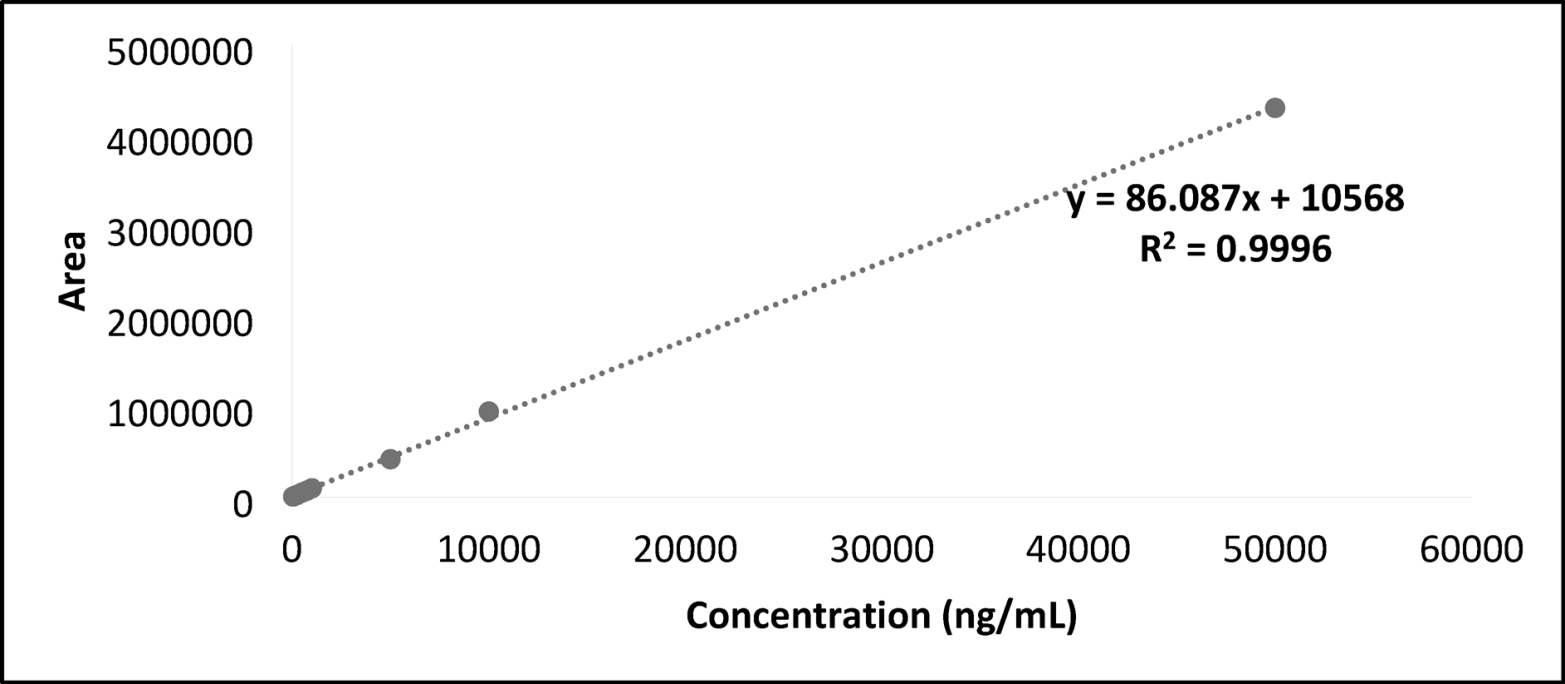
Calibration Graph for QCT using the Optimized Analytical HPLC method (Mobile phase: A-45% ACN; B-55% Buffer; Flow rate: 0.95 mL/min; Concentration range: 0.05–50 µg/mL).

A QCT concentration versus peak area calibration graph was created with the obtained values summarized in Table S4 (Supplementary Information). The R^2^ value was found to be 0.9996, which is closest to 1 and within the acceptable range, as shown in Fig. 4.

### Robustness

The robustness of the optimized analytical method was evaluated for its endurance and sustainability by deliberately altering the established method’s chromatographic conditions, including the pH of the buffer (±0.2), the mobile phase composition (±2% v/v), the column oven temperature (±2 °C), the wavelength (±2 nm), the injection volume (±2 µL), and the flow rate (±0.1 mL/min). It was observed from Tables S4 and S5 in Supplementary Information that both the Rt and the peak area of QCT changed minimally when the wavelength (373 nm vs. 369 nm) and buffer pH (2.8 vs. 3.2) were tweaked. Whereas altering the injection volume (18 µL vs. 22 µL) produced a significant variation in peak area, it had a minimal effect on the Rt. This demonstrates how accurately the method responds to sample preparation/loading, and it also emphasizes how crucial it is to maintain constant injection volumes for quantitative evaluations. Rt was significantly affected by changes in flow rate (0.85 mL/min vs. 1.05 mL/min), with a higher flow rate producing significantly shorter Rt and a lower flow rate producing longer Rt. As a result, the peak area also changed with flow rate, demonstrating its critical impact on method output. Rt and peak area showed significant variations when the mobile phase ratio was changed (43A 57B vs. 47A 53B), suggesting the importance of preserving the optimum phase composition for repeatable outcomes. Rt and peak area only slightly changed when the temperature of the column oven was changed from 23 to 27°C, indicating that the tested range was rather tolerant to temperature changes.

In summary, the wavelength and mobile phase pH had a minimal impact on responses; the injection volume significantly affected the peak area. Flow rate and mobile phase composition caused notable shifts in both Rt and peak area, while the column oven temperature had only a minor effect on Rt. The method responses changed slightly as a result of deliberate modifications to the optimised method variables. However, the RSD (%) values of all alterations were observed to be consistent and well within the acceptable limits (2%). This demonstrates the robustness, suitability, and applicability of the established method for QCT quantification across different formulations, while ensuring reliable performance despite minor routine variations.

### Precision

The intra-day and inter-day precision of the approach was tested at three different QCT levels: LQC (750 ng/mL), MQC (1250 ng/mL), and HQC (2500 ng/mL), as observed in Table S4 of the Supplementary Information, using six runs of the same vial at different time points. The RSD (%) for peak area at each QCT concentration indicates the precision of the implemented method. All results obtained fell within the recommended range, indicating that the optimized procedure was highly precise. For acceptance requirements, the RSD or Coefficient of Variance (%) values of ≤1% for intra-day and ≤2% for inter-day precision were taken into account.

### Accuracy

Three levels of reference standards (made directly from working standard solution) and test samples (made using the allegation method) were created using the stipulated approach. The accuracy of the analytical approach, expressed in terms of recovery percentage, was used to evaluate how near the obtained value was to the actual value. The % RSD values were found to be well within the limits of acceptance (98 to 102%), according to Table S4 (Supplementary Information).

### 24 Hours Bench-top stability

The recovery of the standing standard solution or freshly prepared samples after 24 hours was found to be quite similar with RSD (<2%), and a similarity index of 1.000 between the freshly prepared and benchtop samples was all within the acceptable limits, as represented in Table S4 (Supplementary Information). The bench-top stability study’s similarity index indicates that the mobile phase and sample solution remain stable for 24 hours^57^.

### Stress-induced degradation studies

Following stress exposure according to above mentioned methodology, the QCT samples were neutralized where required, diluted with the standard diluent, and further analyzed using the validated RP-HPLC method.

Under accelerated conditions, QCT showed significant degradation, particularly in strong acid and alkaline environments. Exposure to 1 N HCl resulted in recoveries below quantifiable limits, with nearly complete degradation (99.76%), indicating extreme instability under highly acidic conditions when heat is applied. Additionally, a notable shift in Rt to 3.686 minutes was observed in this condition, which may suggest either altered analyte behaviour or the presence of a co-eluting degradation product under strong acid and thermal stress. Similarly, in 1 N and 0.1 N NaOH, the drug exhibited recoveries below quantifiable limits (BQL) due to complete degradation or instrument sensitivity limits under these conditions, confirming pronounced instability in basic media, irrespective of base strength or exposure duration. Mild acidic conditions (0.1 N HCl) produced moderate degradation (51.79%), while oxidative stress led to 48.45% degradation, indicating that degradation accelerates in the presence of heat and oxidizing agents. Moist heat exposure also caused significant degradation (52.37%), likely due to hydrolytic effects facilitated by water.

Contrarily, under ambient bench-top conditions, QCT demonstrated relatively better stability. In the presence of 0.1 N HCl and 30% H₂O₂, the compound showed minimal degradation and high assay recovery. However, the drug remained highly unstable in both 1 N and 0.1 N NaOH at room temperature, with recoveries below the quantifiable limit (BQL), highlighting its sensitivity to alkaline conditions even in the absence of heat. Thermal stress under dry heat (80 °C for 12 hours) resulted in moderate degradation (16.66%) with 93.34% recovery, indicating that the compound is relatively stable in its solid form at elevated temperatures. Photolytic degradation under UV exposure resulted in a recovery of 87.24%, indicating modest degradation compared to chemical and thermal stresses.

**Fig. 5:**
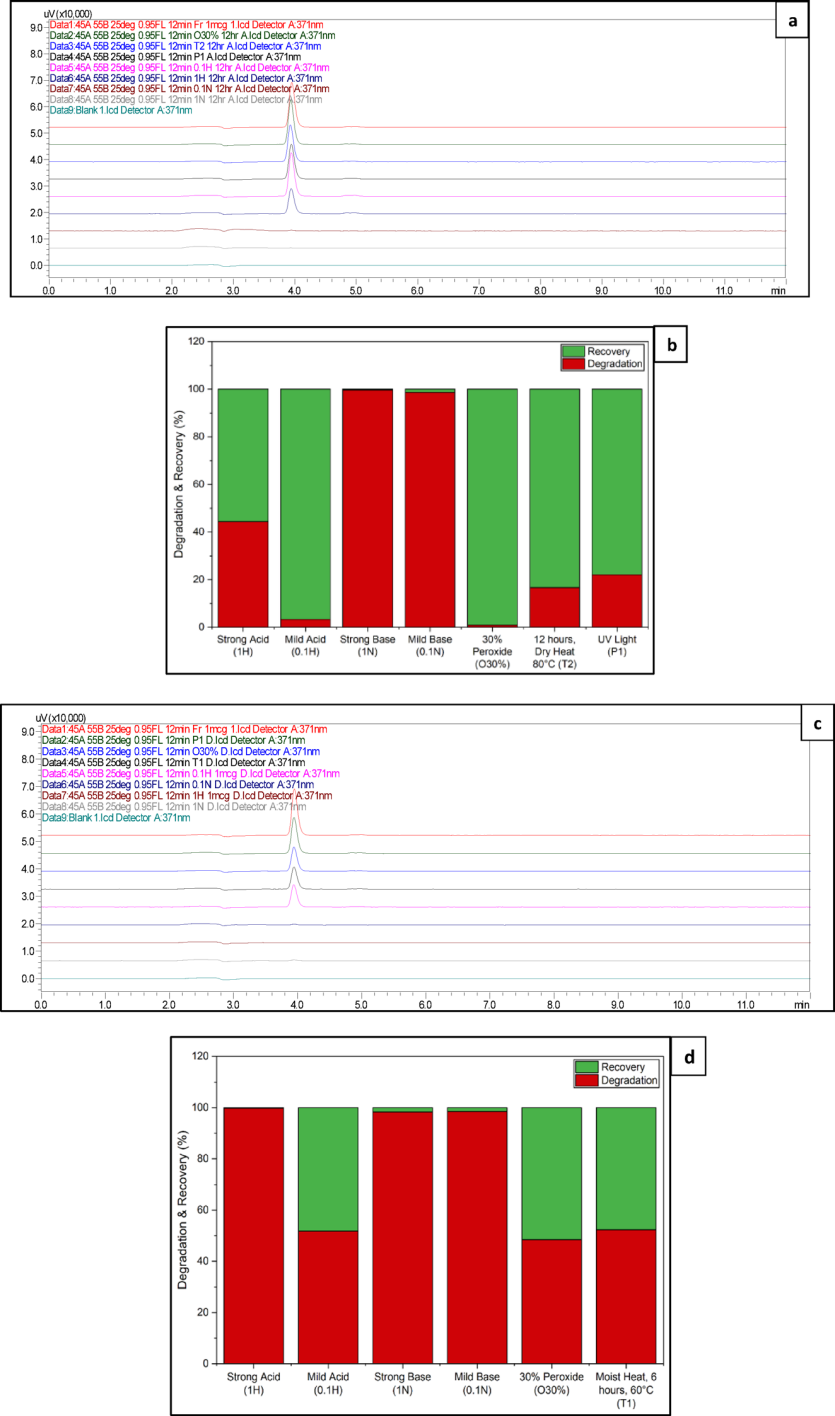
HPLC overlay Chromatograms of QCT in stress-induced degradation studies and bar graphs of recovery and degradation % of QCT in Bench-top and Accelerated conditions. **a** HPLC chromatogram of Bench-Top degradation (12 h at room temperature) showing stacked baseline comparisons of QCT exposed to different stressors along with blank (Data 9), and freshly prepared control (Data 1—Fr); **b** Bar-graph of recovery and degradation % of QCT in Bench-Top degradation condition. **c** HPLC chromatogram of Accelerated degradation (6 h at 60 °C oven temperature) showing stacked baseline comparisons of QCT exposed to different stressors along with blank (Data 9), and freshly prepared control (Data 1—Fr). **d**. Bar-graph of recovery and degradation % of QCT in the Accelerated degradation condition.

Throughout all other conditions, the Rt of QCT remained consistent (3.93-3.95 minutes), and no interfering peaks from degradation products were observed (Fig. S9 of Supplementary Information). The single exception was the notable shift under accelerated acidic stress (1 N HCl at 60 °C), which could warrant further investigation to rule out co-elution or structural modification of the analyte. This anomaly aside, the method consistently demonstrated specificity and a means to precisely measure QCT in the presence of its degradation byproducts. A summary of all the stressors, along with their responses, is represented below in Table 4, along with their representations in Fig. 5.

**Table 4:** Comparative evaluation of QCT degradation under accelerated (60 °C, 6 h) and Bench-Top (RT, 12 h) stress Conditions: % Theoretical accuracy and % degradation Profiles.

Stress condition (sample code)	Bench-top degradation (12 h exposure at room temperature)			Accelerated degradation (6 h exposure at 60 °C oven temperature)			Interpretation
	Ret. Time (min)	% Accuracy	% Degradation	Ret. Time (min)	% Accuracy	% Degradation	
1 N HCl: Strong Acid (1 H)	3.94	58.94%	44.36%	3.68	*BQL*	99.76%	Strong acid significantly accelerates degradation with heat. Negative recovery at 60 °C implies complete instability. Degradation at RT is comparatively mild.
0.1 N HCl: Mild Acid (0.1 H)	3.94	96.39%	3.26%	3.94	49.65%	51.79%	Observed to be relatively stable at RT in the presence of low acid concentration. Heat causes moderate degradation and lower recovery.
1 N NaOH: Strong Base (1 N)	3.93	*BQL*	99.62%	3.94	*BQL*	98.24%	Highly degraded under both basic and heated conditions. Consistently negative recovery confirms severe instability in a strong base irrespective of heat.
0.1 N NaOH: Mild Base (0.1 N)	3.93	*BQL*	98.60%	3.94	*BQL*	98.45%	Strongly unstable and degraded even at low base concentration. Stability unaffected by heat.
30% H₂O₂: Oxidative stress (O30%)	3.93	98.96%	0.94%	3.94	54.25%	48.45%	Oxidative degradation is minimal at RT, but significantly increases at 60 °C. The accuracy drops under heat, suggesting oxidation accelerates with temperature.
Moist Heat: 6 h, 60 °C (T1)	—			3.94	48.76%	52.37%	Moist heat causes significant degradation, with 52.37% recovery. The presence of water likely facilitates hydrolytic breakdown.
Dry Heat: 12 h, 80 °C (T2)	3.93	93.34%	16.66%	—			Moderate degradation even after exposure to high temp (80 °C) for 12 h in its solid state. Accuracy still acceptable; drug remains relatively stable in powder form.
UV Light (P1)	3.94	87.24%	22.05%	—			Light-induced degradation is modest compared to thermal/acid/base.

**(*BQL: Below Quantifiable Limit*)**.

In conclusion, the implemented RP-HPLC method is validated as a robust, specific, and reliable stress-testing method capable of distinguishing intact QCT from its degradation products across a range of ICH-recommended stress conditions.

### Application of the developed Validated RP-HPLC method

#### Preparation and characterization of QCT-AgMOF formulation

After the preparation of AgMOFs, the DLS reports demonstrate that the mean particle size of AgMOFs was found to be 178.4 ± 1.08 nm, the average Zeta Potential was -26.567 ± 0.493, and the average polydispersity index was observed to be 0.229 ± 0.01, which supports the monodispersed property of the particle size in the formulation as shown in Fig. S10 of the Supplementary information. The reported physical characteristics indicate that the AgMOFs meet the requirements of a topical drug delivery application for Diabetic wound therapeutics.

Furthermore, SEM (Scanning Electron Microscopy) was carried out to assess the surface morphological features of the prepared AgMOF structure. Imaging was performed using a Zeiss EVO 18 SEM operated at 10.00 kV accelerating voltage. Fig. S11 of the Supplementary information exhibits a high-magnification SEM visualization of the AgMOFs system (35.00 K magnification, with a working distance of 8 mm). For the purpose of antibacterial application in the treatment of DFU, the high surface area and nanoparticulate size observed in the SEM images are likely to be desirable for improved contact with bacterial cells. The granular morphology also confirmed that MOFs with distinct nanoscale characteristics were successfully formed, which could result in formulations with improved drug loading and optimum drug release profiles.

#### Entrapment efficiency (EE%) and drug loading (DL%) of QCT in AgMOFs

**Fig. 6:**
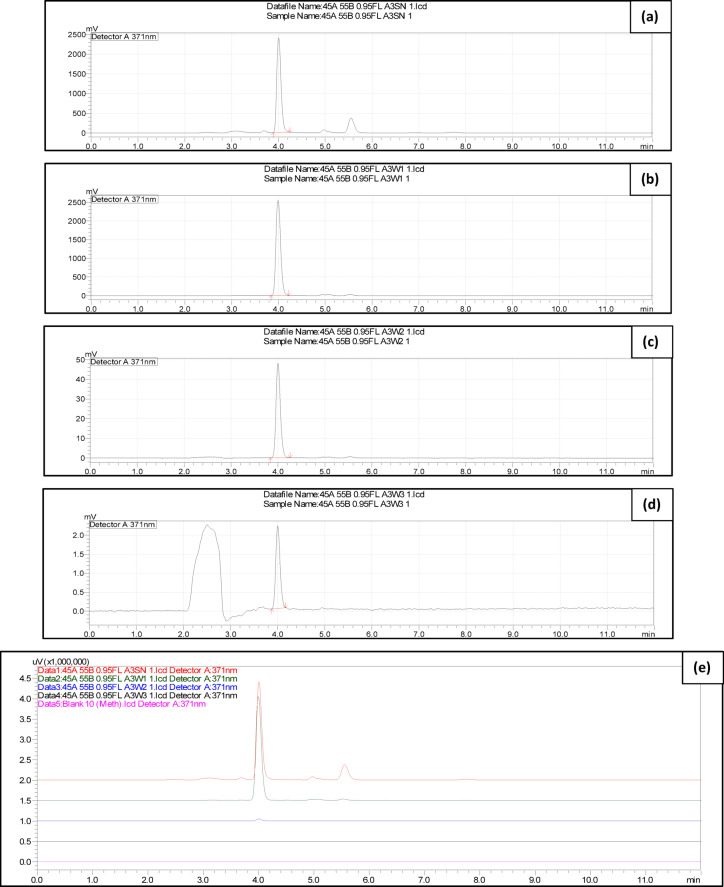
HPLC chromatograms containing the unentrapped drug of the AgMOF system after 24 h drug loading for the evaluation of DL% and EE%. (**a**) Direct supernatant (A3SN-Data 1); (**b**) Supernatant from first wash (A3W1-Data 2); (**c**) Second wash (A3W2-Data 3); (**d**) Third wash (A3W3-Data 4); (**e**) Data comparison chromatogram, where the free drug samples (Data 1, 2, 3 and 4) are stacked with the blank Methanol (Data 5).

QCT loaded in the AgMOFs system was found to be precisely quantified utilizing the devised RP-HPLC method, proving its applicability for a wide range of QCT-loaded complex drug-delivery systems. The method’s specificity was confirmed by the chromatograms, which displayed a clear peak in the specific Rt window of the drug, as seen in Fig. 6. The total unentrapped QCT (free drug) concentration in the supernatant samples was found to be 389712.49 ng/mL (389.71 µg/mL), according to the HPLC analysis of the 2 mL aliquot as shown in Table S6 (Supplementary Information). Hence, by further extending this value to the entire 30 mL solution, where a total of 30,000 µg of drug was incorporated into a nanocarrier formulation weighing 30 mg, the total QCT concentration was determined to be:

$${\mathrm{Total}} QCT {\text{concentration in the}} QCT - AgMOFs = \frac{389.71 \times 30}{2} = 5845.68 \mu g$$

After separation of the unencapsulated QCT, the amount of free QCT recovered from the supernatant was 5,845.68 µg. Based on this, the calculated entrapment efficiency (EE%) was found to be 80.52%, indicating efficient encapsulation of the drug within the nanocarrier matrix. The corresponding drug loading (DL%) was determined to be 44.59%, reflecting a high proportion of drug content relative to the total weight of the formulation. These findings imply that QCT is effectively encapsulated by the current formulation approach and can be precisely quantified using the developed RP-HPLC method.

## Greenness assessment of developed method

The AGREE tool was used to appraise the sustainability of the developed RP-HPLC method for the estimation of QCT in the QCT-AgMOF formulation. Sample preparation, reagent sustainability, waste production, energy use, and solvent toxicity, specifically the selection of ACN and acidified water as mobile phase components, were all taken into account in this assessment. Optimization of the procedure reduced overall reagent consumption and waste, in alliance with green analytical chemistry principles.

**Fig. 7:**
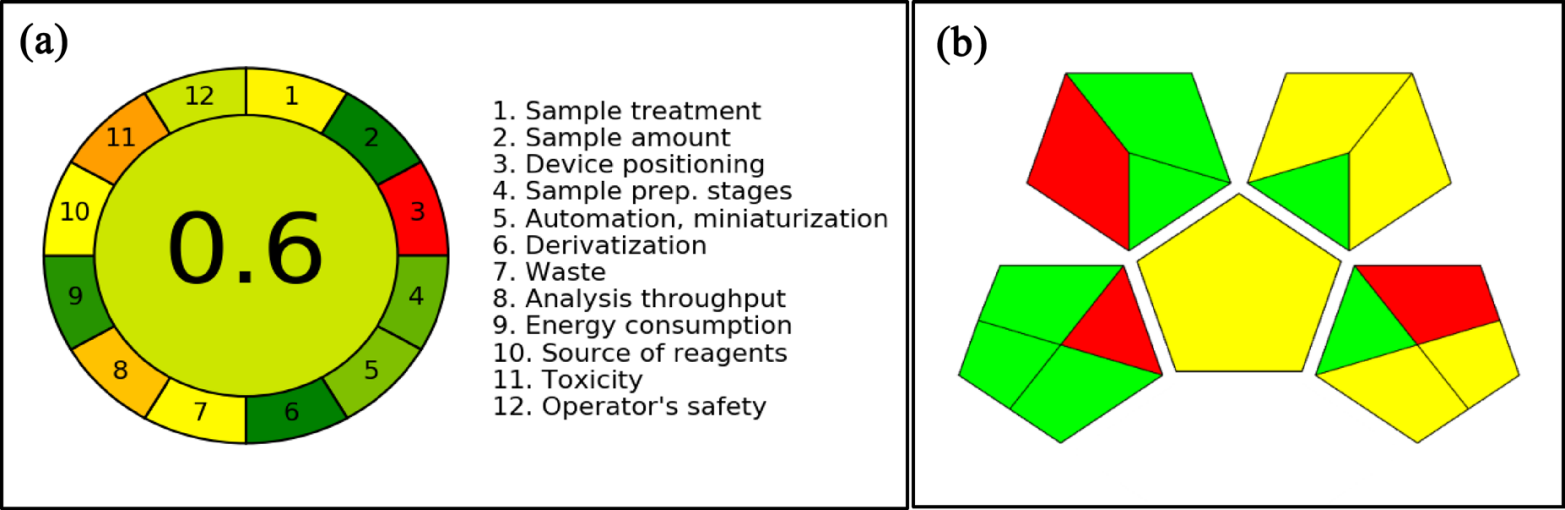
Greenness Assessment of Developed RP-HPLC Analytical Method illustrations. (**a**) AGREE pictogram of the implemented RP-HPLC method for estimation of QCT; (**b**) ComplxGAPI pentagram representing the Greenness of the implemented RP-HPLC method for the Qualitative analysis of QCT.

An AGREE score of 0.6 and the ComplexGAPI pentagram in Fig. 7 indicate that the DoE-based HPLC analytical method for QCT evaluation is environmentally acceptable. The petrochemical background of ACN and the minimal energy usage throughout the 12-minute run and processing of the sample are reflected in its mild greenness profile. The method’s advantage of requiring fewer optimization trials reinforces both sustainability and efficiency in pharmaceutical and nanomaterial analysis, further supporting the concepts of GAC.

## Discussion

In comparison to the previously published methods for QCT analysis, the developed RP-HPLC method exhibits a number of noteworthy advantages over their disadvantages, as represented in Table 5 below. Compared to methods that employ acetic or phosphoric acid, which usually do not have adequate buffering capacity, the approach that employs TEA as a buffering component achieves excellent pH stability and significantly minimizes Tf across both basic and acidic analytes. In contrast to lower pH conditions (such as pH 2.6), adjusting the mobile phase to pH 3.0 with OPA creates a less hostile environment, thereby extending column lifespan and reducing the likelihood of silica dissolution while still effectively suppressing silanol activity to produce sharp, well-resolved peaks^58^. The use of a straightforward, two-component mobile phase, which consists solely of ACN and a buffered aqueous phase, not only improves repeatability and simplifies preparation, but it also makes scalability easier for regular quality control applications. Furthermore, in various analytical circumstances, the selection of the C18 column also plays a crucial role in reproducibility, prolonged column life, and consistent chromatographic performance. The new approach is significantly superior to previously reported HPLC methods for QCT assessment due to these characteristics, which, when combined, render it less susceptible to baseline noise or corrosion and extremely resilient for routine, long-term use.

As demonstrated by its application with QCT-loaded AgMOFs, this study supports the implementation of the sustainable RP-HPLC method as a reliable analytical tool for measuring QCT in complex formulations. Furthermore, an AGREE assessment score of 0.6 supports the claim of a sustainable, cost-effective and environmentally friendly method, probably attained by producing less waste, shortening the overall run time, using a more balanced mobile phase, simplifying sample preparation, saving energy and using the system more efficiently^59,60^. The superior specificity, sensitivity, and adaptability of this approach can be used in future research on HPLC quantification of QCT delivery systems via oral, topical, and transdermal routes. This improves clinical translation by enabling accurate drug quantification, formulation optimization, quality assurance, and regulatory compliance. In future prospects, the applicability of this method can be further expanded beyond *in vitro* studies to *in vivo* and clinical research by implementing bioanalytical method development for detecting QCT in biological matrices^48^. This will enable reliable pharmacokinetic, safety, and therapeutic monitoring studies in various QCT-loaded formulations.

**Table 5:** Compilation of advantages and disadvantages of published HPLC methods for QCT Estimation.

Sr. no.	Method Description	Advantages	Disadvantages	Reference
1.	C18 column (250 × 4.6 mm, 5 μm); Acetonitrile:2% acetic acid (40:60, v/v, pH 2.6); Column temperature of 35 ℃; Flow rate of 1.3 mL/min; LOD: 1.22 ng/mL	Rapid, ICH-validated, simultaneous detection	• Column lifespan may be shortened by a highly acidic mobile phase (pH 2.6) • Total run time per injection is 18.5 min, which is relatively long and consumes more mobile phase and resources, further increasing operational costs and impacting the Greenness of the method. • Requires temperature control throughout • Acetic acid can cause baseline noise and corrosion • Less buffer capacity, so less pH stability compared to TEA buffer • Not optimal for basic analytes and may cause peak tailing	Ang. et al. ^31^
2.	C18 column; Water: ACN: MeOH (55:40:5) with 1.5% acetic acid; Flow rate of 1.0 to 1.3 mL/min; LOD: 0.046 µg/mL	High sensitivity, fast elution, ICH validated	• Complex mobile phase is difficult to prepare and reproduce • Requires access to diode array detection (DAD), which may not be available in all HPLC systems • Not robust for routine analysis and is not able to resolve the tailing phenomenon • No buffer for pH control, so Rt can drift • Not ideal for long-term reproducibility	Carvalho, D. et al. 60
3.	C18 column; Buffer phase: H₂O (0.1% formic acid, A); Organic phase: MeOH and ACN, (40:15v/v), B), Mobile phase (A: B), 40:60 (v/v); Flow rate of 0.8 mL/min	Selective, ICH validated for plant extracts	• Gradient methods require more equilibration and system suitability checks • Longer Rt of 7.58 min • Its practicality for samples with much higher or lower analyte concentrations without further sample dilution or concentration alterations may be limited because the validated method exhibited linearity in a very narrow concentration range (5–17.5 µg/mL for QCT).	Pandiyan, R. et al. 61
4.	C18 column (250 × 4.6 mm, 5 μm); Gradient elution; Mobile phase consisted of (A) ACN: (B) 0.1% Phosphoric acid (aqueous buffer); Flow rate was 1.0 mL/min, Injection volume was 10 µL; Column maintained at ambient temperature and Run time was 27 min	Optimized for QCT glycosides present in onion; high specificity for plant matrices; validated for onion cultivars.	• The total run time per injection is 27 min, which is relatively long and consumes more solvent systems, further increasing operational costs and environmental impact • Gradient elution increases solvent use (Higher organic solvent consumption up to 60%) and equilibration time • Phosphoric acid (pH ~ 1.8 to 2.2) may shorten column lifespan, poor pH control and lack of ionic strength, eventually might affect reproducibility. • Not salt-free (if phosphate buffer used)	Phattanaphakdee, W. et al. 62
5.	C18 column; MeOH: Water (60:40, v/v); Flow rate of 0.8 mL/min; Injection volume of 50 µL	Simple, linear, good accuracy	• MeOH can increase backpressure (flammability) when compared to the use of ACN • No buffer, so poor pH control and lack of ionic strength, eventually affecting reproducibility. • Not suitable for pH-sensitive analytes • Limited selectivity for closely eluting peaks • 50 µL exceeds the recommended injection volume, which can overload the column, reduce resolution, and worsen the overall peak shape.	D’Mello et al. ^63^
6.	C18 column (150 × 4.6 mm, 5 μm); Mobile phase: 0.1% TFA in water and ACN (35:65 v/v), isocratic; Column temp: 25 °C; Flow rate: Not specified; Detection at 255 nm; Injection volume: 25 µL; LOD: 0.04–0.1 µg/mL (media dependent); LLOQ: 0.12–0.31 µg/mL.	Linear, selective, sensitive, precise and accurate; Method ICH validated in methanol, FaSSGF, FeSSGF, PBS pH 7.4.	• Flow rate not mentioned • Validation requires the availability of standard and blank media • Slightly lower LOD/LLOQ in certain media • A high percentage of ACN can shorten column life and further increase method cost due to organic solvent consumption.	Permana, A. D. et al. ^64^

## Conclusion

A robust, sustainable, RP-HPLC method was developed and refined for the assessment of QCT within various pharmaceutical formulations, such as Ag-MOFs, as discussed in this research. Clinically, QCT has the highest therapeutic dose range in oral formulations compared to other dosage forms. According to a study conducted by Sarah *et al.*, oral supplementation of QCT (500-1000 mg/day dose) leads to peak plasma concentrations between 100 and 500 nmol/L or approximately 30-150 ng/mL ^65^. Hence, the established linearity range of the developed method (0.05-50 μg/mL) completely encompasses the expected plasma concentrations after oral (500-1000 mg/day), intravenous, and even topical/transdermal doses, where systemic absorption is typically low (Table 1). Given the method’s LOD (40.27 ng/mL) and LOQ (122.03 ng/mL), it can accurately and reproducibly quantify QCT in post-dose plasma concentrations, ensuring robustness and sensitivity suitable for therapeutic monitoring. A three-level Box-Behnken response surface design of experiment was implemented in the method development to attain a superior, feasible and reproducible chromatographic performance. The developed RP-HPLC method using acidified water (0.1% v/v TEA, pH 3.0 adjusted with OPA) and ACN (55:45% v/v) via an isocratic elution demonstrated excellent chromatographic performance. The method provided sharp and well-defined peaks with consistent peak area, ensuring reliable estimation of QCT in complex matrices. These results confirm that the optimized mobile phase composition and chromatographic conditions are suitable for sensitive, accurate, and precise analysis of QCT, making this method robust for routine quality control applications. This work presents an important case study demonstrating the application of RP-HPLC technology for assessing QCT in various formulations. The optimized approach exhibited linearity, sensitivity, accuracy, precision, and robustness over a wide range of concentrations when verified in accordance with ICH requirements. Considering that it separated QCT from degradation products, stress testing in acidic, alkaline, oxidative, and thermal environments validated the method’s potential to indicate stability. The ability to separate QCT from the polymeric matrix further demonstrated the method’s accuracy, and intra- and inter-day percentage RSD of less than 2% attests to its precision. Hence, QCT loaded in the AgMOF nanoparticulate system was successfully and precisely quantified in this work, owing to the devised HPLC method, subsequently making it easier to determine the drug loading and entrapment efficiency. Everything being considered, this validated HPLC approach can be used for routine standard quality control and dose assessment in a variety of pharmaceutical and nutraceutical commodities. It may also be modified to estimate the amount of anti-inflammatory and antioxidant flavonoids structurally similar to QCT in a variety of formulation matrices. It is an efficient means for analysis and industrial quality assurance, considering its dependability, specificity, and adaptability, which could further contribute to the design and enhancement of sophisticated drug delivery systems for deployment in the foreseeable future.

## Supplementary Information

Below is the link to the electronic supplementary material.

Supplementary Material 1

## Data Availability

All data generated or analyzed during this study are included in this published article and in its Supplementary Information file.

## Abbreviations

QCT: Quercetin
MeOH: Methanol
ACN: Acetonitrile
ANOVA: Analysis of variance
DLS: Dynamic light scattering
