# Supplementary material for: Quality-by-design based RP-HPLC analytical method for the estimation of quercetin in silver-metal organic frameworks (AgMOF): a Box-Behnken optimization approach
Source: Sci Rep. 2025 Dec 18;16:2. doi: 10.1038/s41598-025-32452-6 (PMC12764978; doi:10.1038/s41598-025-32452-6)
Supplement: Supplementary file 1 — Supplementary Material 1 [file 41598_2025_32452_MOESM1_ESM.docx]

**Supplementary Information**

**Quality-by-Design based RP-HPLC Analytical method for the Estimation of Quercetin in Silver-Metal Organic Frameworks (AgMOF): A Box-Behnken Optimization Approach**

Anoushka Mukharya, Srinivas Mutalik^*^

Department of Pharmaceutics, Manipal College of Pharmaceutical Sciences

Manipal Academy of Higher Education, Manipal 576104, Karnataka, India

*** Corresponding author:**

Dr Srinivas Mutalik

Principal and Professor

Manipal College of Pharmaceutical Sciences

Manipal Academy of Higher Education

Manipal 576104, Karnataka State, India

Email: ss.mutalik@manipal.edu

**Figures**


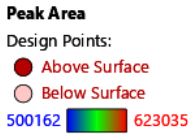

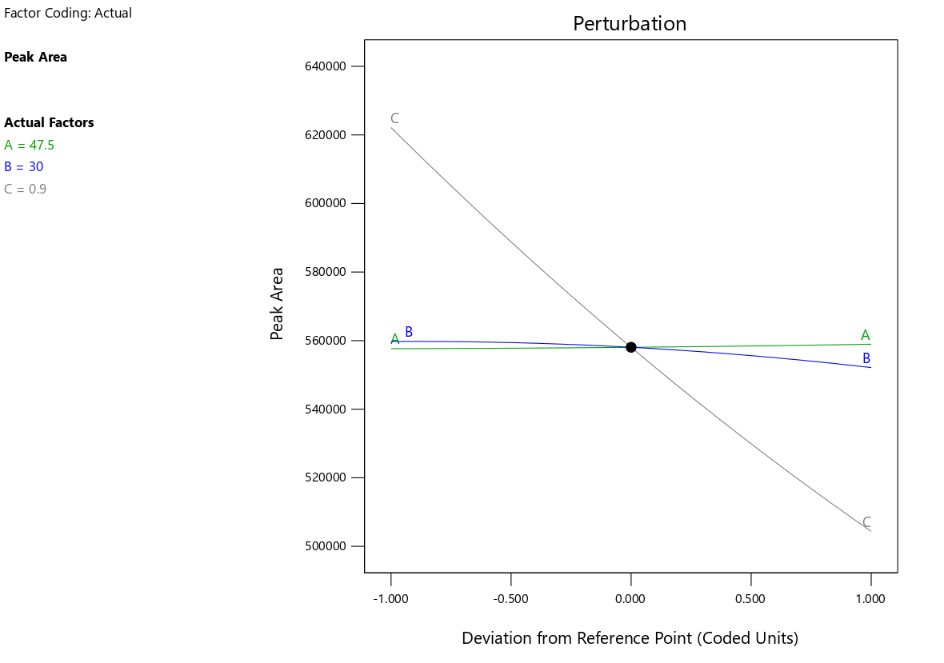

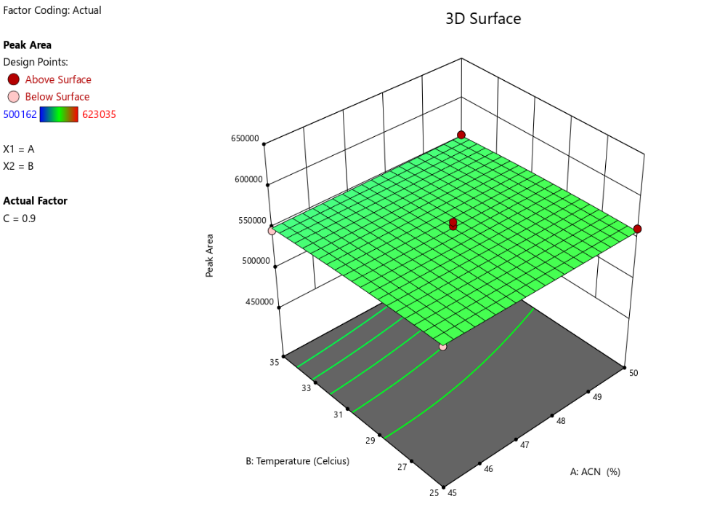

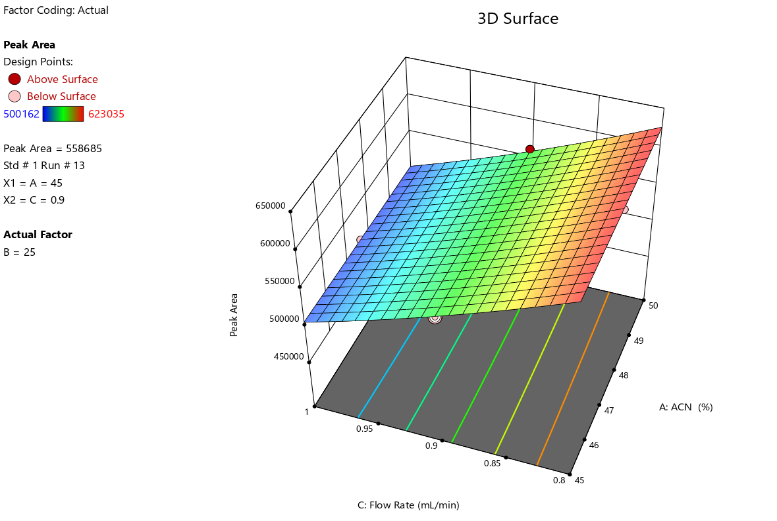

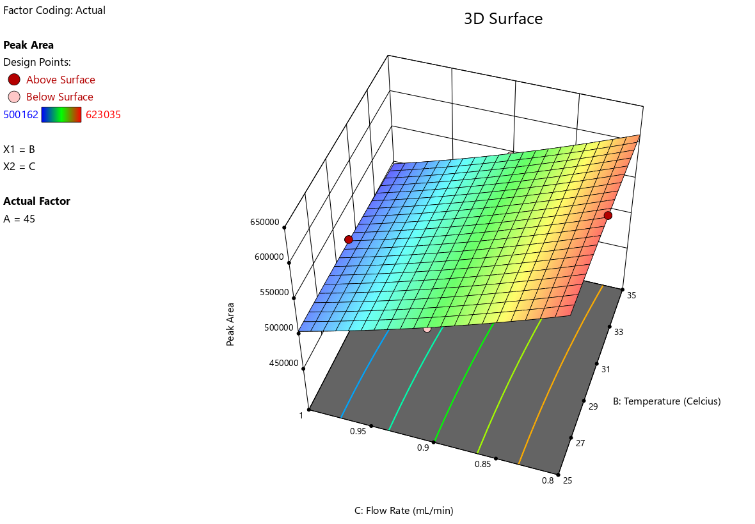


**b**

**e**

**c**

**d**

**a**

**Figure S1. Perturbation Plot and 3D Surface Diagrams of Peak Area analysis.**

**[a]** **Perturbation Plot representing the effect of Independent Variables on the analysed Peak Area (R1);**

**[b] Key representing the colour codes and Peak Area range for the 3D surface diagrams;**

**[c] 3D Surface graph for the effect of Factors A and B on Peak Area;**

**[d] 3D Surface graph for the effect of Factors A and C on Peak Area;**

**[e] 3D Surface graph for the effect of Factors B and C on Peak Area.**

**
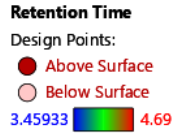
**
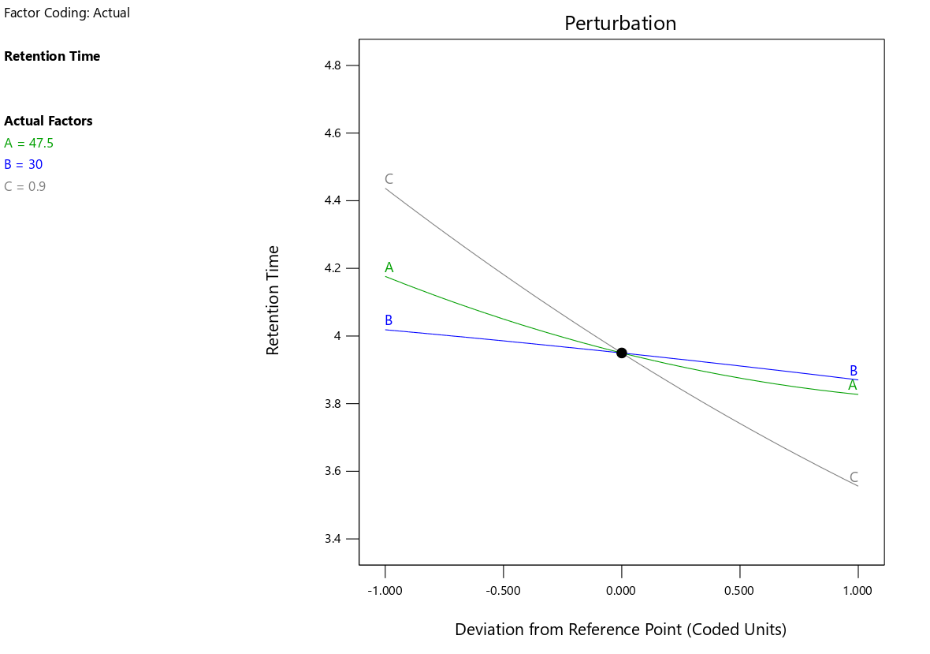

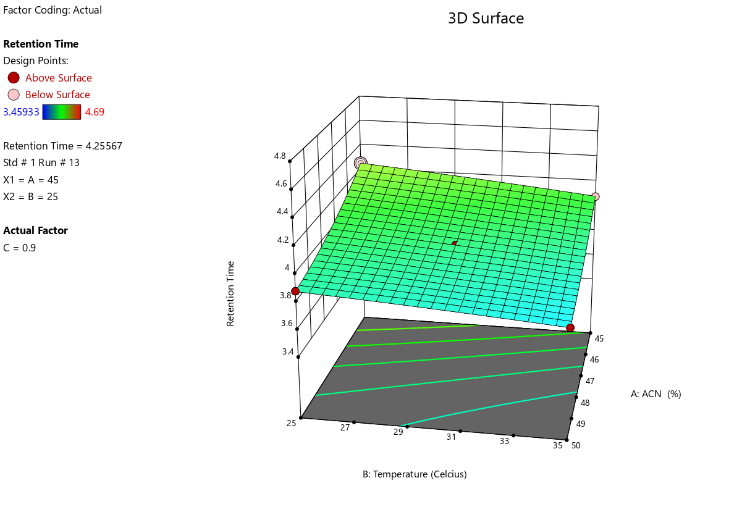

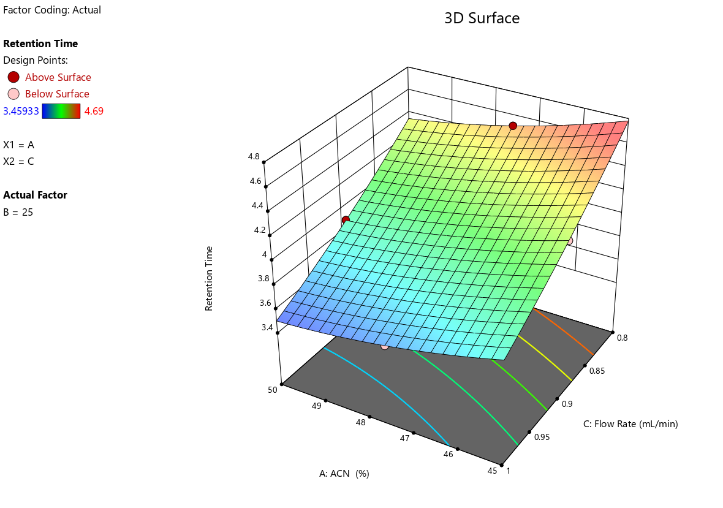

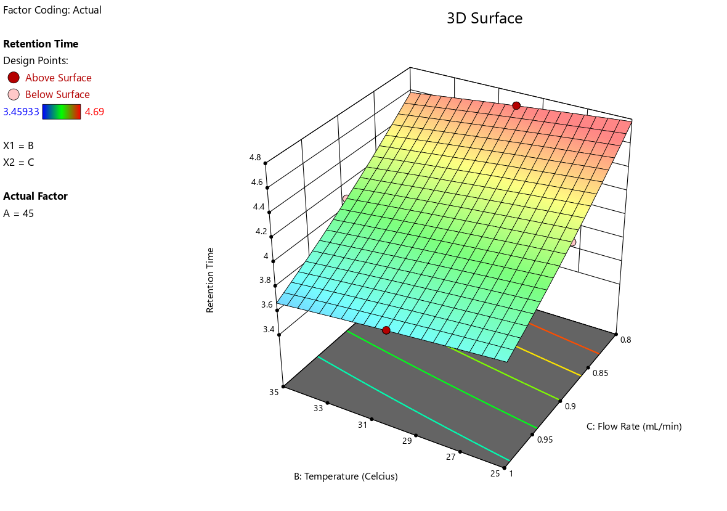


**c**

**d**

**b**

**a**

**e**

**Figure S2. Perturbation Plot and 3D Surface Diagrams of Retention Time analysis.**

**[a] Perturbation Plot representing the effect of independent variables on the analysed peak Retention Time (R2);**

**[b] Key representing the colour codes and peak Retention Time range for the 3D surface diagrams;**

**[c] 3D Surface graph for the effect of Factors A and B on peak Retention Time;**

**[d] 3D Surface graph for the effect of Factors A and C on peak Retention Time;**

**[e] 3D Surface graph for the effect of Factors B and C on peak Retention Time.**


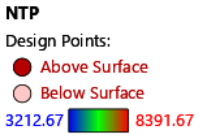

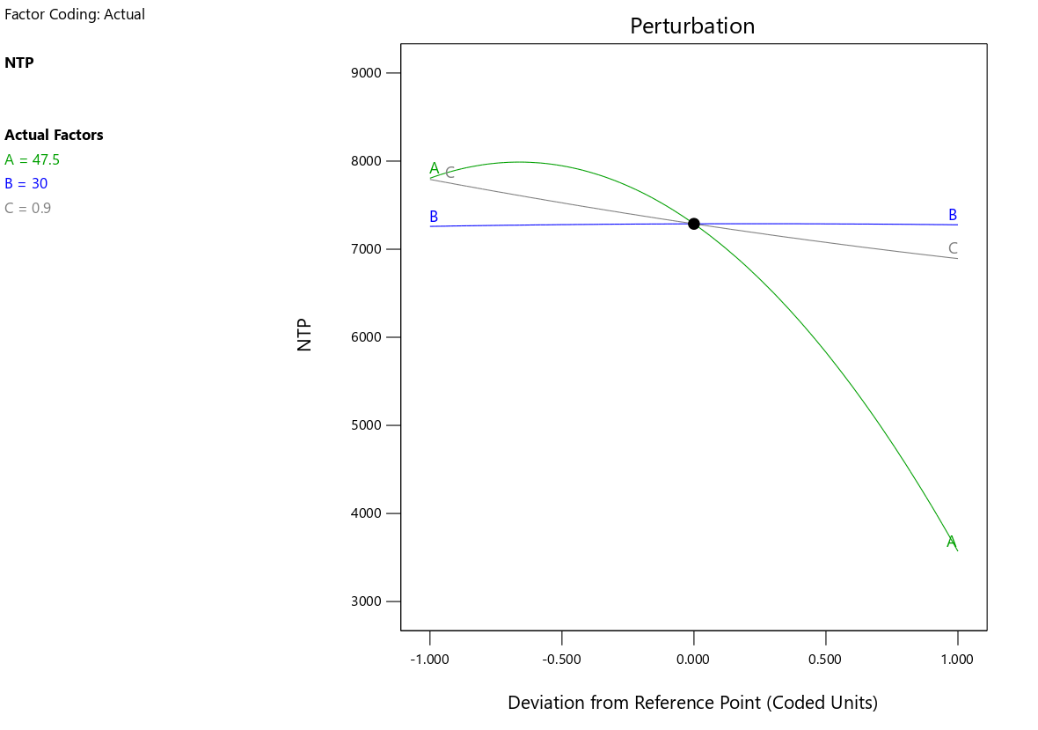


**b**

**a**


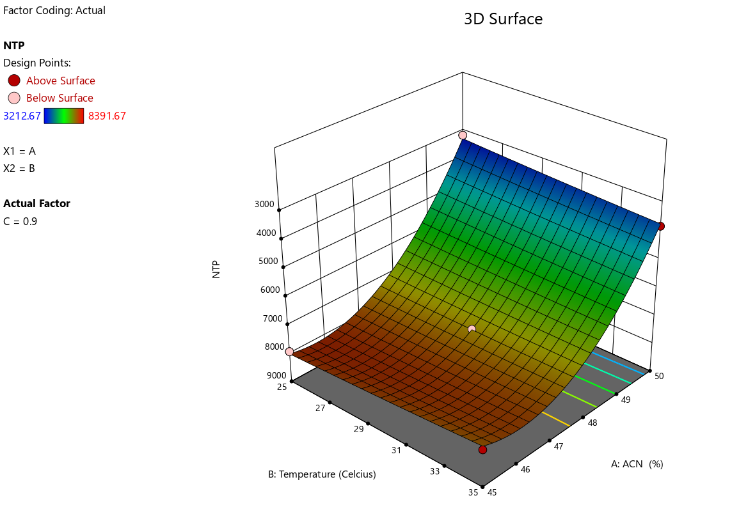

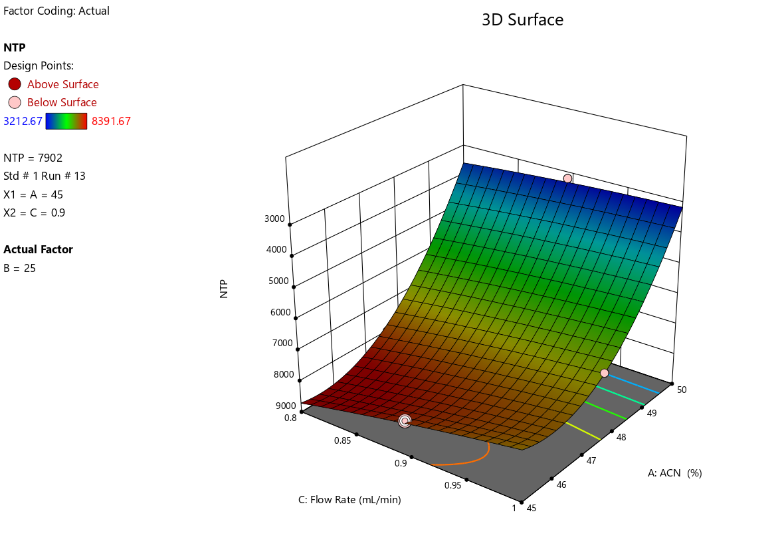

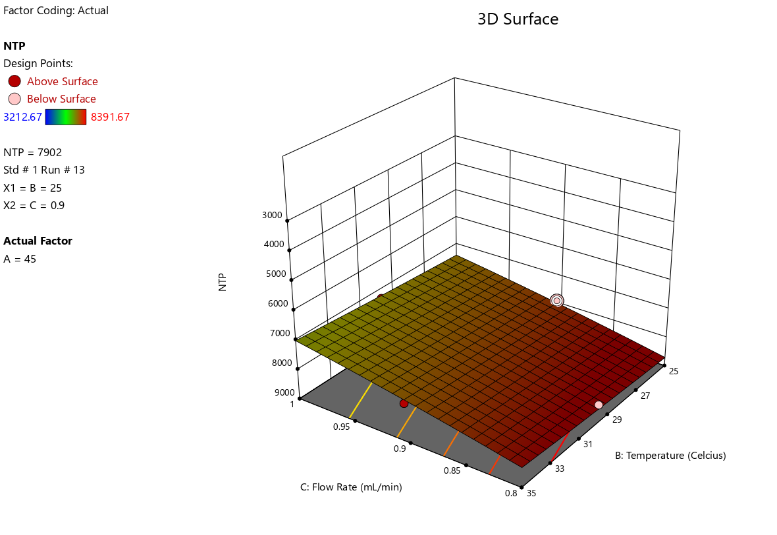


**e**

**d**

**c**

**Figure S3. Perturbation Plot and 3D Surface Diagrams of Theoretical Plates (NTP) analysis.**

**[a]** **Perturbation Plot representing the effect of independent variables on the analysed Theoretical Plates (NTP) (R3);**

**[b] Key representing the colour codes and theoretical plates (NTP) range for the 3D surface diagrams;**

**[c] 3D Surface graph for the effect of Factors A and B on Theoretical Plates (NTP);**

**[d] 3D Surface graph for the effect of Factors A and C on theoretical plates (NTP);**

**[e] 3D Surface graph for the effect of Factors B and C on theoretical plates (NTP).**


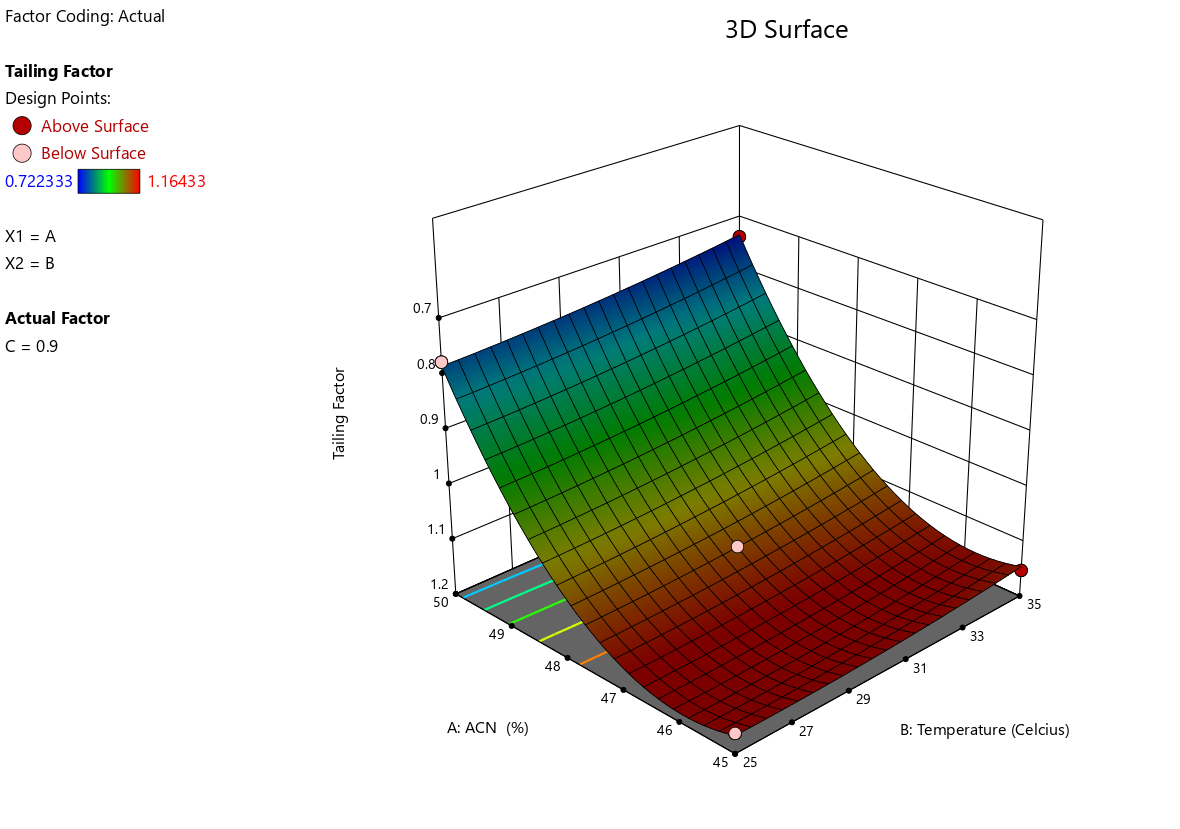

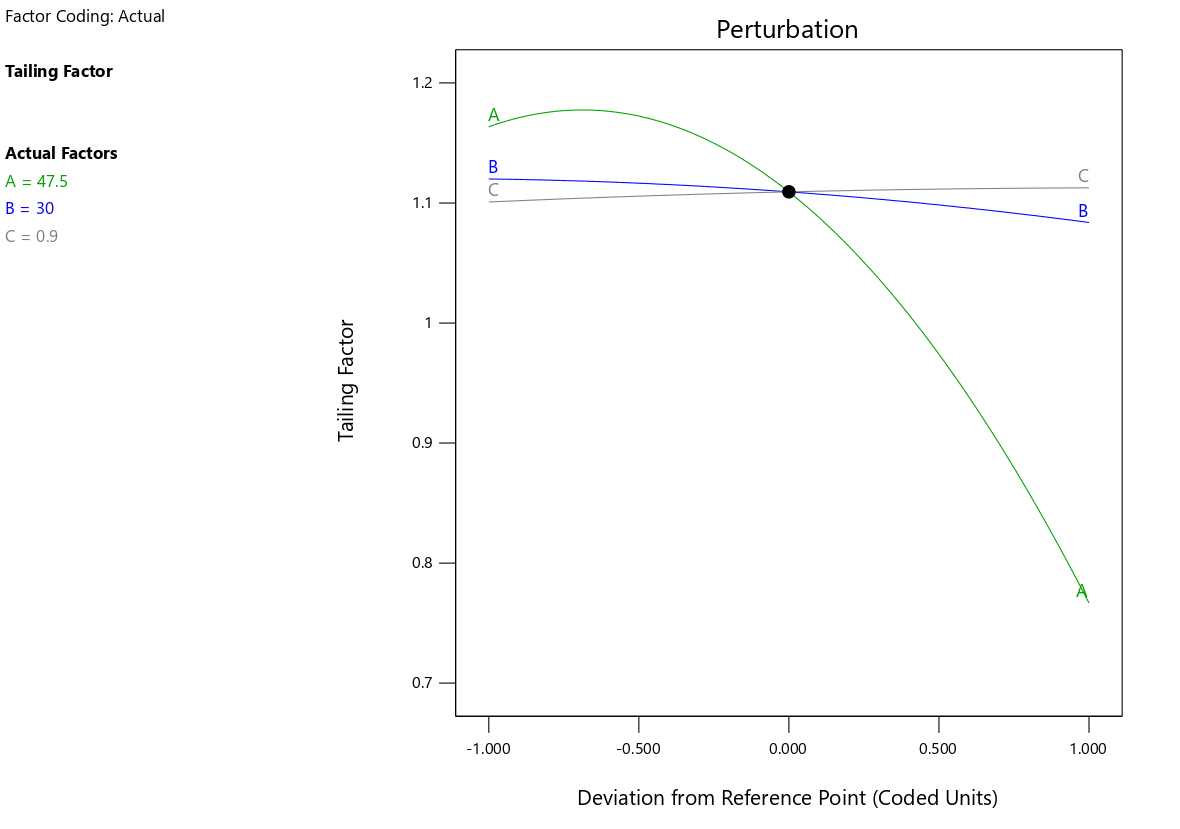


**a**

**b**


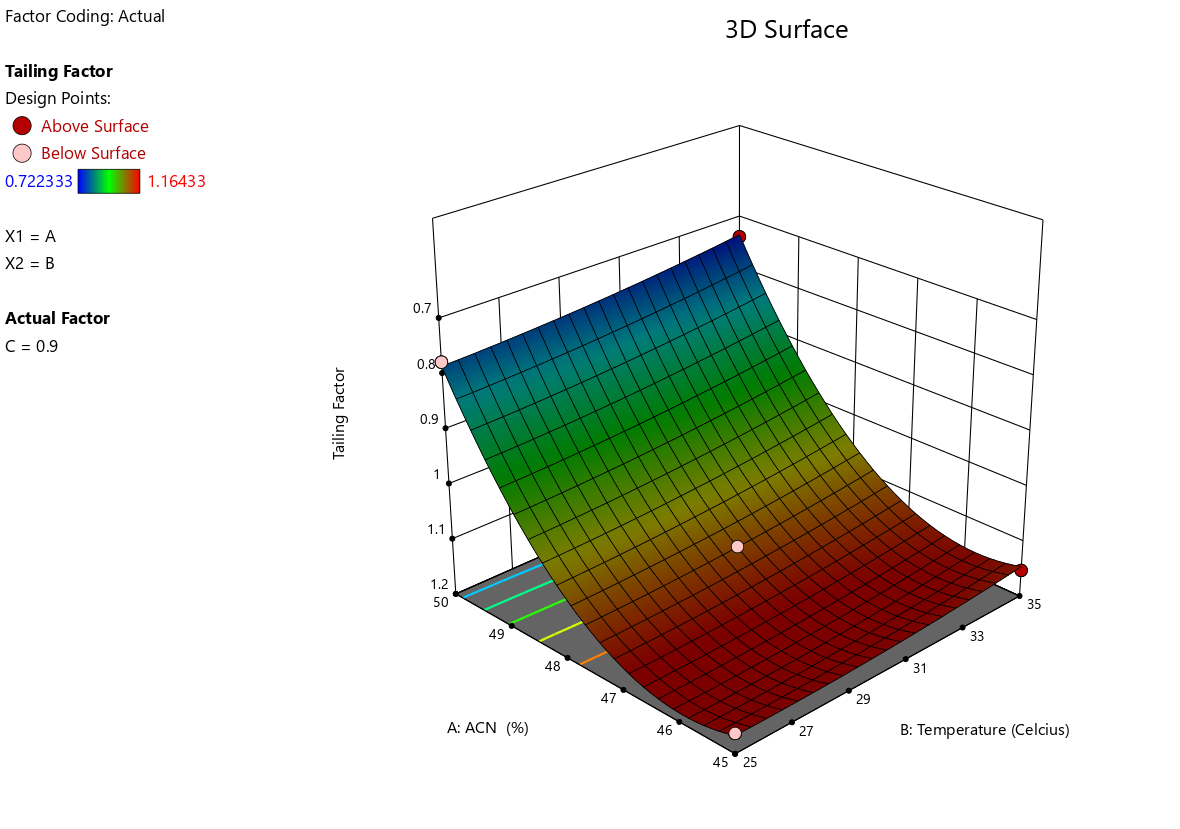

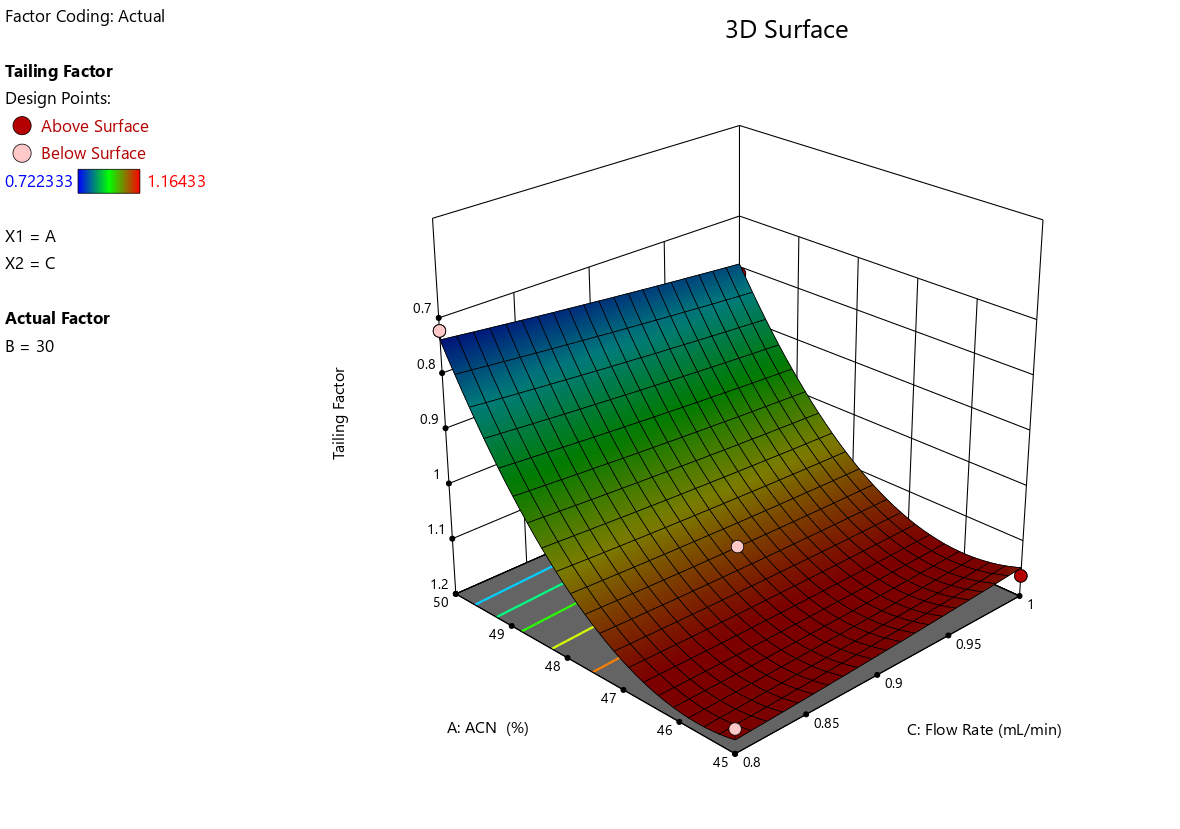

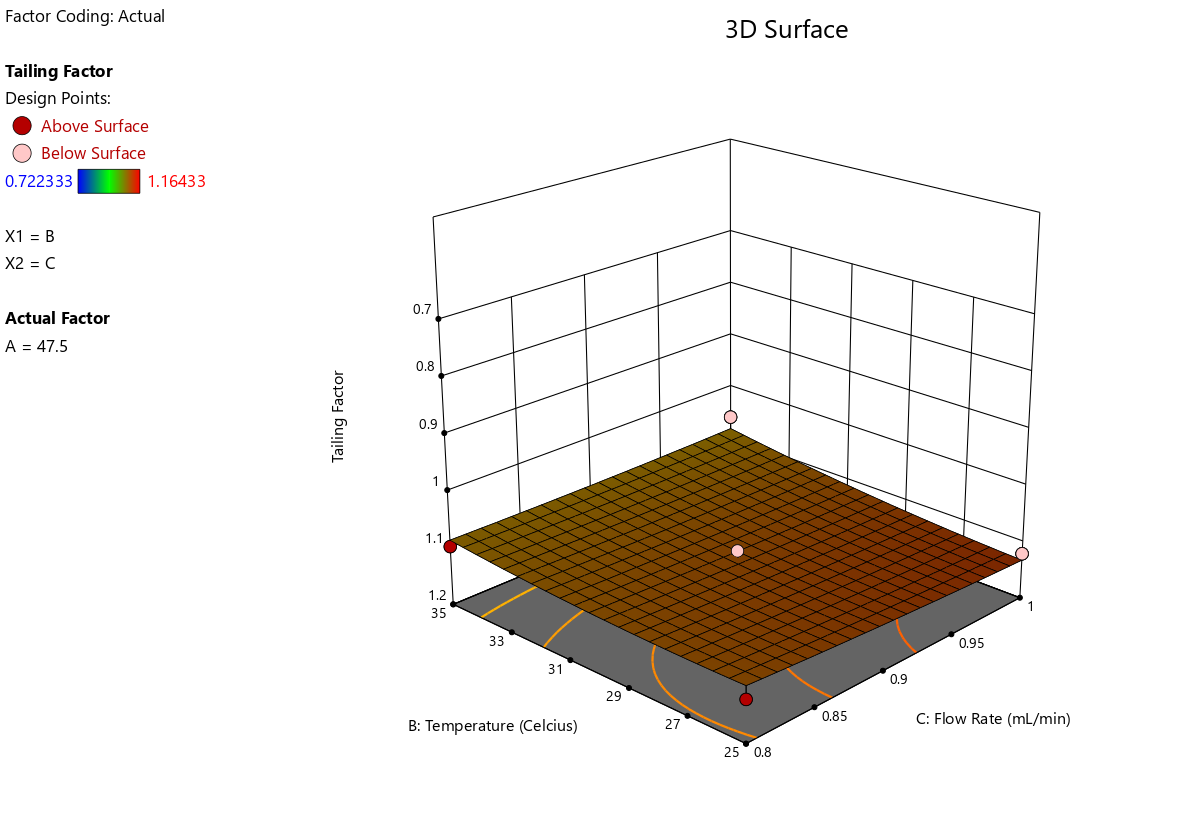


**e**

**d**

**c**

**Figure S4. Perturbation Plot and 3D Surface Diagrams of Tailing Factor analysis.**

**[a] Perturbation Plot representing the effect of independent variables on the analysed Tailing Factor (R4).**

**[b] Key representing the colour codes and Tailing Factor range for the 3D surface diagrams.**

**[c] 3D Surface graph for the effect of Factors A and B on Tailing Factor.**

**[d] 3D Surface graph for the effect of Factors A and C on Tailing Factor.**

**[e] 3D Surface graph for the effect of Factors B and C on Tailing Factor.**

**Run 5**

**Run 6**

**Run 4**

**Run 3**

**Run 1**

**Run 2**

**Run 7**

**Data Comparison (Same Baseline)**

**Data Comparison (Base-shifted)**

**Figure S5. All 7 trial run peaks complementary to the given Table S2 parameters, and the data comparison peaks for all 7 runs stacked differently based on the Box-Behnken design of Experiment.**

**
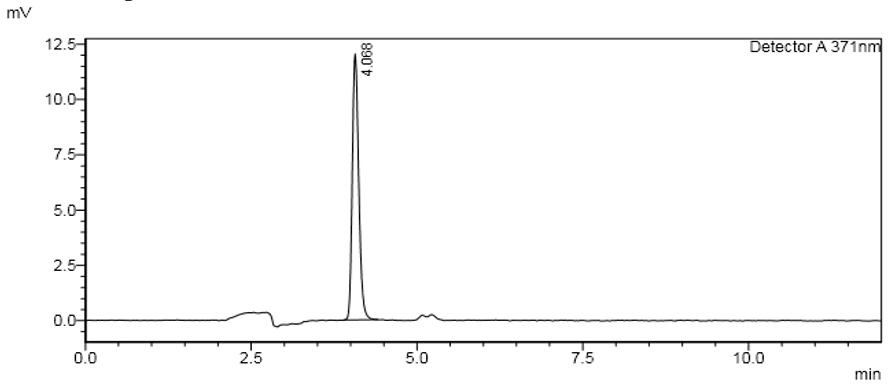
**
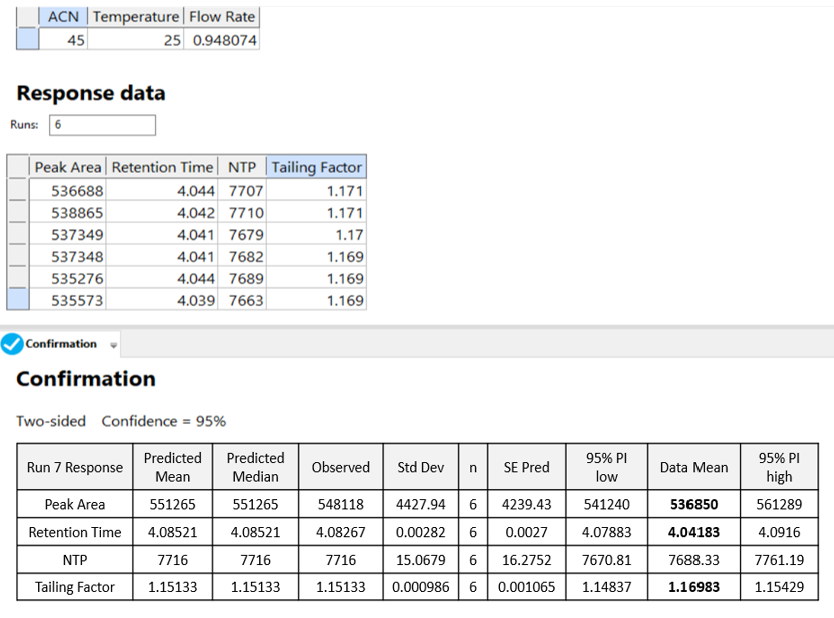


**a**

**b**

**c**

**Figure S6.** **RP-HPLC Analytical Method Predicted Run 7 (Solution 1) Experimental Confirmation.**

**[a] Response Confirmation data with a 95% Confidence level by repeating the solution 6 times (n=6), using Box-Behnken design of Experiment;**

**[b] Optimized Run 7 (Solution 1) response HPLC Chromatogram;**

**[c] Overlay HPLC chromatogram of optimized solution (n=6) with stacked baselines, further validating its system suitability.**


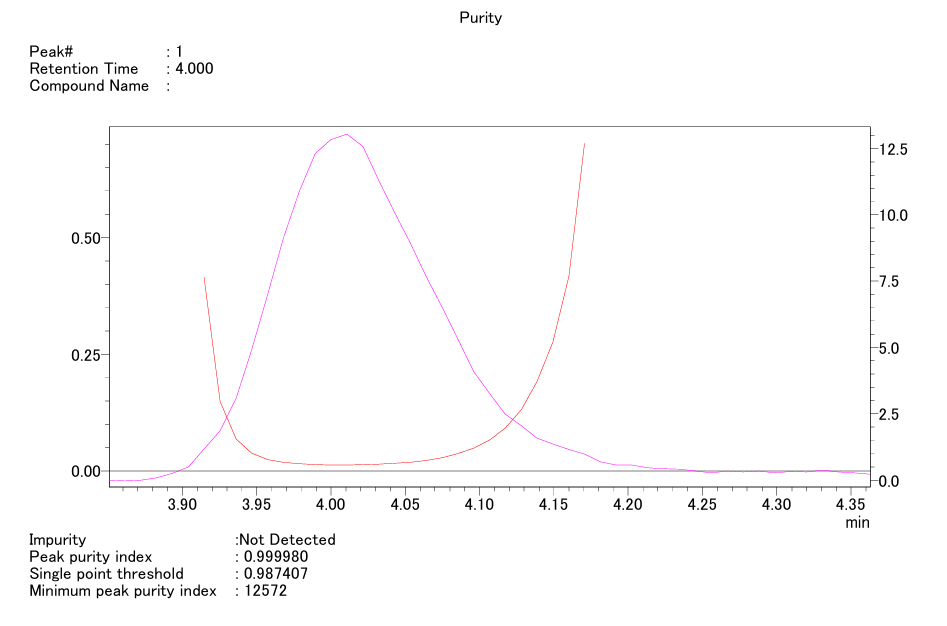


**a**


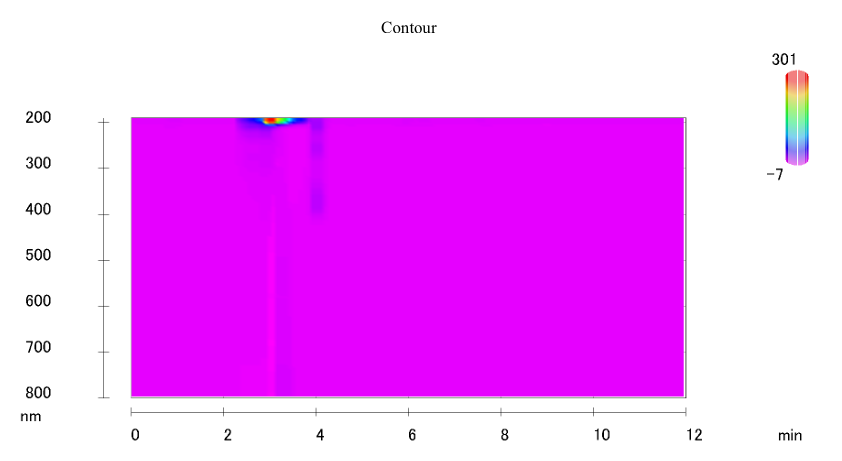


**b**


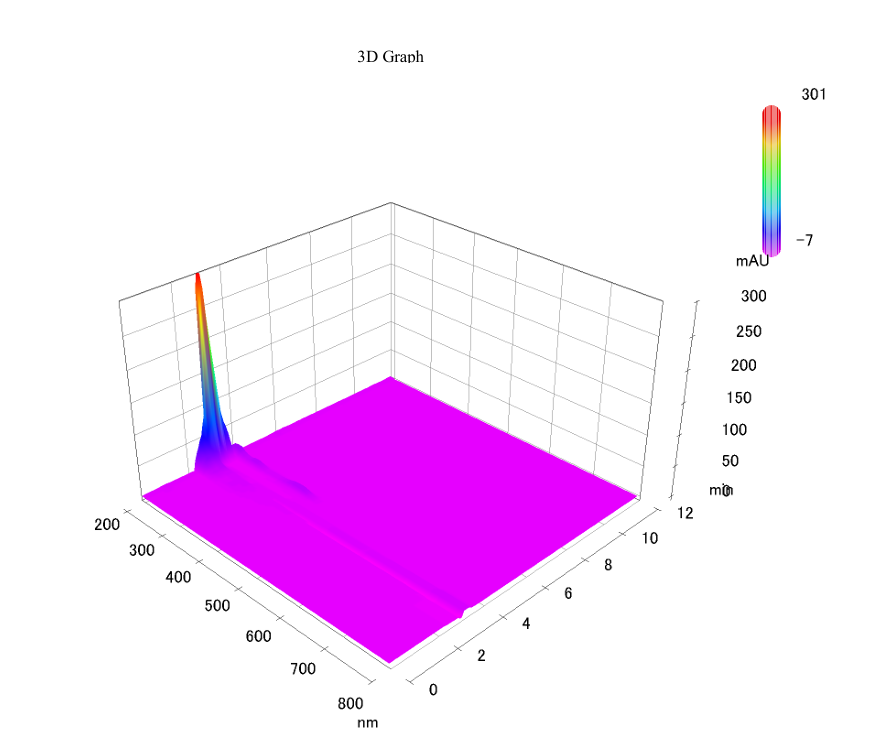


**c**

**Figure S7. Peak Purity and Spectrum Analysis of 1 μg/mL QCT sample run using optimized method.**

**[a] Peak purity plot at retention time 4.000 min, illustrating high purity index (0.999980) and absence of detected impurities;**

**[b] Contour plot showing absorbance intensity across 200–800 nm at 12 minutes acquisition time, visualizing the retention and spectral distribution of the analyte peak;**

**[c] 3D absorbance surface plot, representing wavelength-specific absorption and elution profile of the analyte for comprehensive purity assessment.**

**a**

**b**

**Figure S8. PDA multiwavelength overlay spectrum of 1 μg/mL QCT sample run using optimized method.**

**[a] Overlaid chromatogram at multiple wavelengths (211-571 nm) demonstrating spectral homogeneity for the analyte peak at ~ 4.0 min, supporting wavelength selection at 371nm;**

**[b] UV-Visible absorption spectrum of the analyte (200-700 nm), confirming characteristic maxima and method wavelength selection at 371nm.**

**
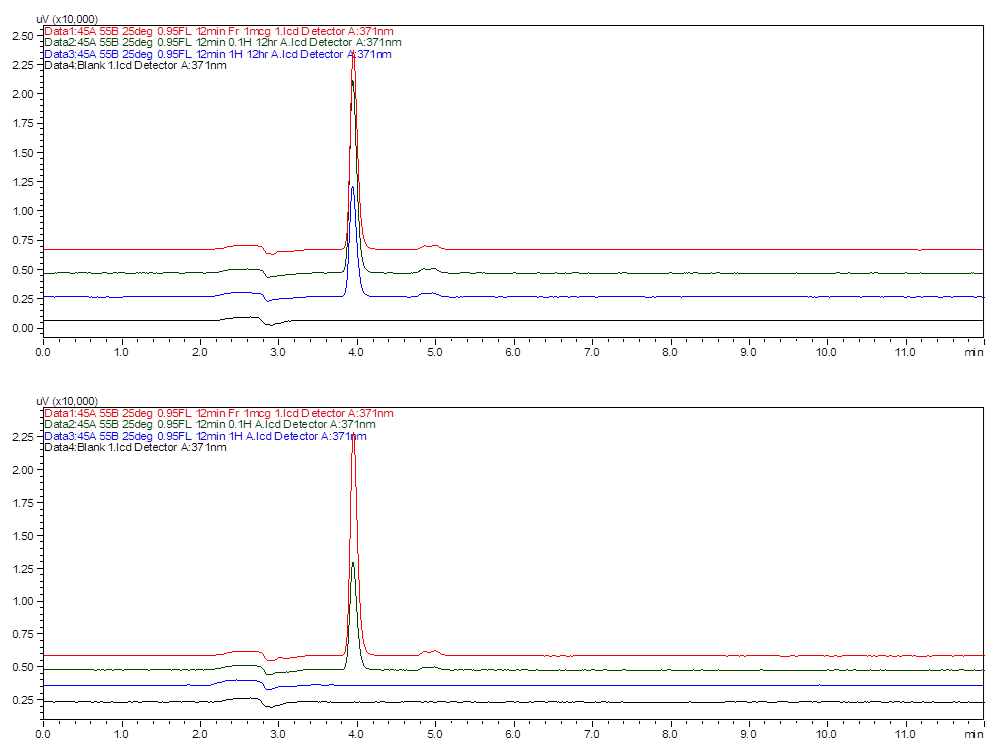
**

**a**

**Acid (Strong and Mild HCl)** **Accelerated Degradation (6 hours exposure at 60°C temperature)**

**Acid (Strong and Mild HCl) Bench-Top Degradation (12 hours exposure at room temperature)**

**
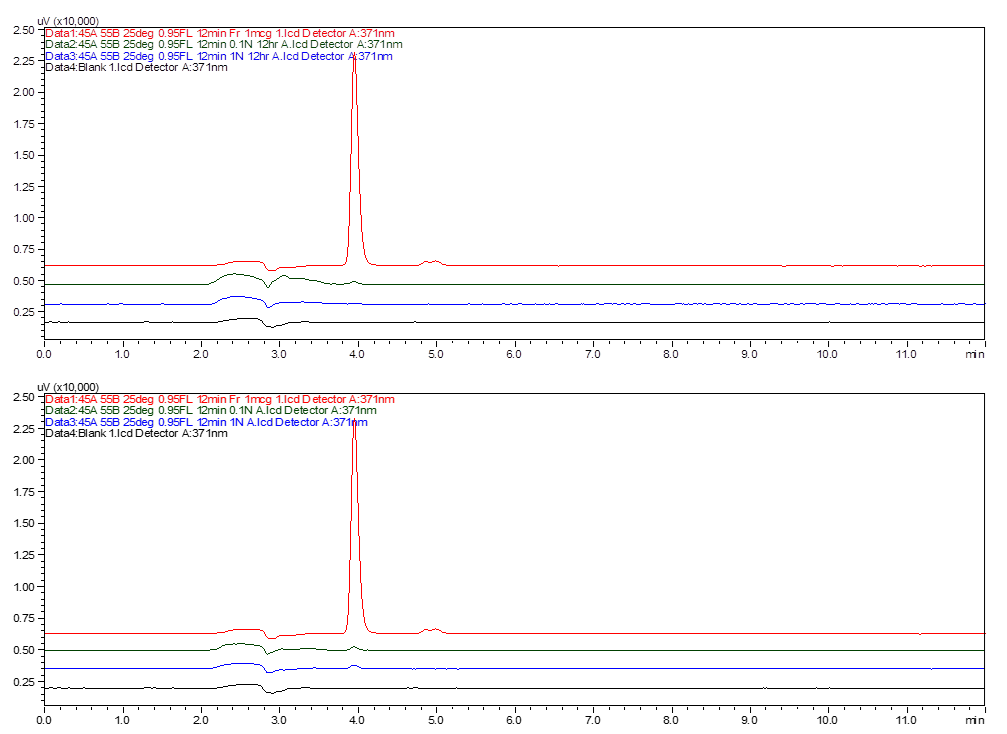
**

**Base (Strong and Mild NaOH) Accelerated Degradation (6 hours exposure at 60°C temperature)**

**Base (Strong and Mild NaOH) Bench-Top Degradation (12 hours exposure at room temperature)**

**b**

**
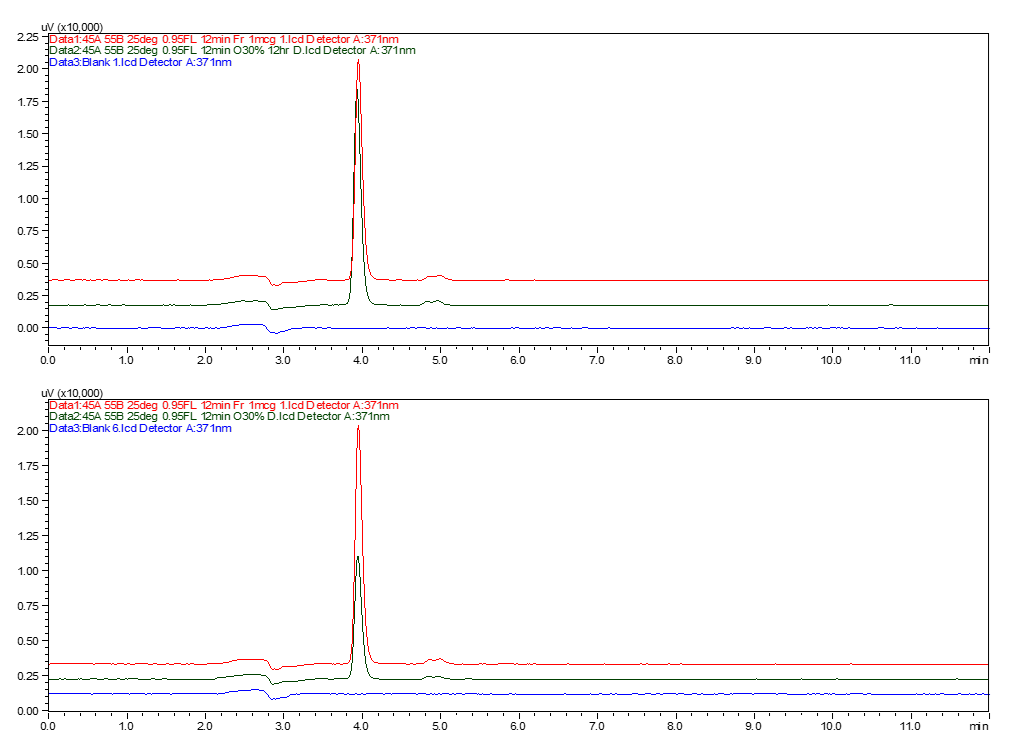
**

**Oxidative (H_2_O_2_) Accelerated Degradation (6 hours exposure at 60°C temperature)**

**Oxidative (H_2_O_2_)** **Bench-Top Degradation (12 hours exposure at room temperature)**

**c**

**
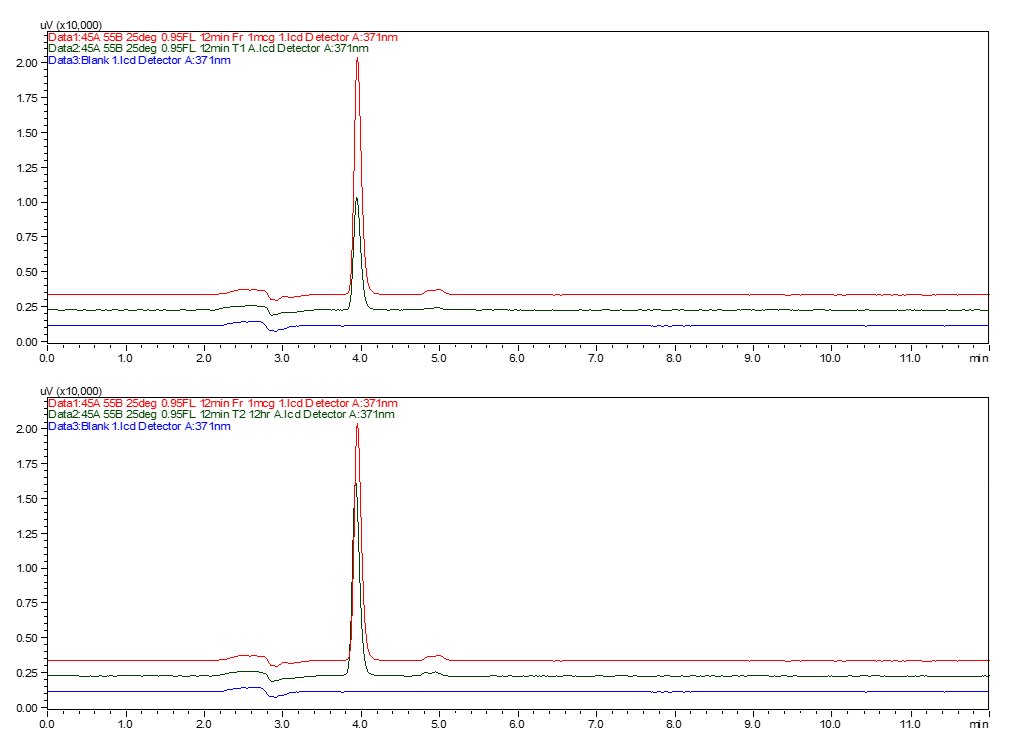
**

**Dry Heat (80°C) Bench-Top Degradation (12 hours exposure at room temperature)**

**Moist Heat Accelerated Degradation (6 hours exposure at 60°C temperature)**

**d**

**UV Light Bench-Top Degradation (12 hours exposure at room temperature)**

**e**

**Figure S9. HPLC chromatograms of Bench-Top and Accelerated Stress Degradation studies showing stacked baseline comparisons of QCT exposed to different stressors along with blank, and freshly prepared control (Data 1 - Fr).**

**[a] Acid (1H-Strong and 0.1H-Mild HCl) Stress Exposure;**

**[b] Base/Alkaline (1N-Strong and 0.1N-Mild NaOH) Stress Exposure;**

**[c]** **Oxidative (O30%-H_2_O_2_) Stress Exposure;**

**[d]** **Moist Heat (T1-6 hours, 60°C) and Dry Heat (T2-12 hours,** **80°C) Stress Exposure;**

**[e] UV Light (P1) 12 hours Stress Exposure.**


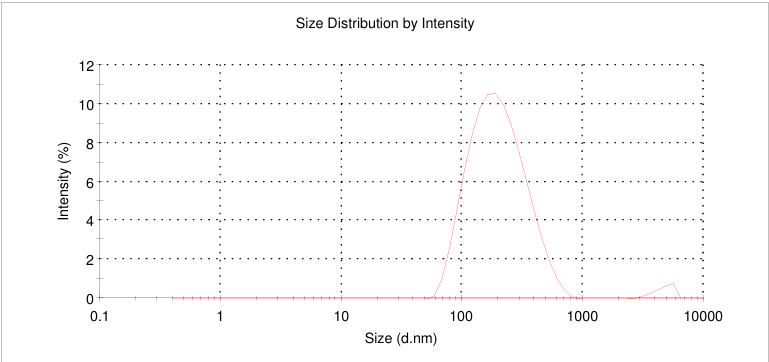


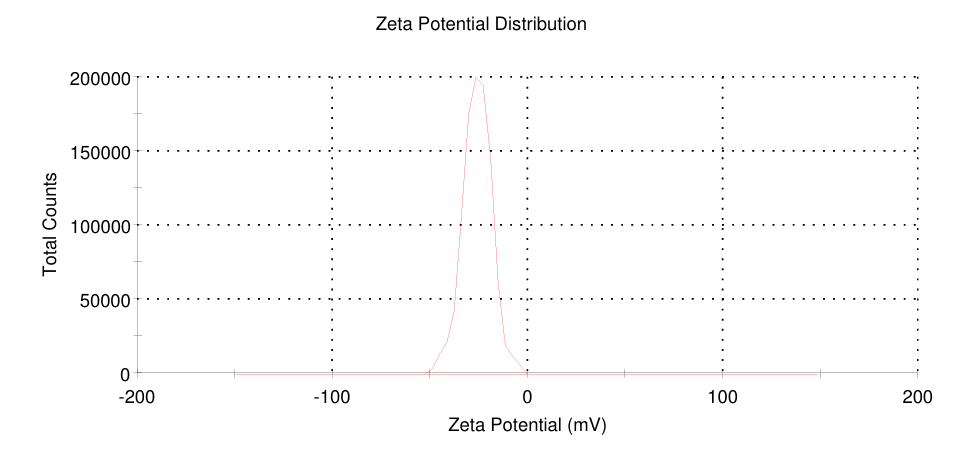


**Figure S10.** **Particle size and Zeta Potential distribution graphs of AgMOFs**


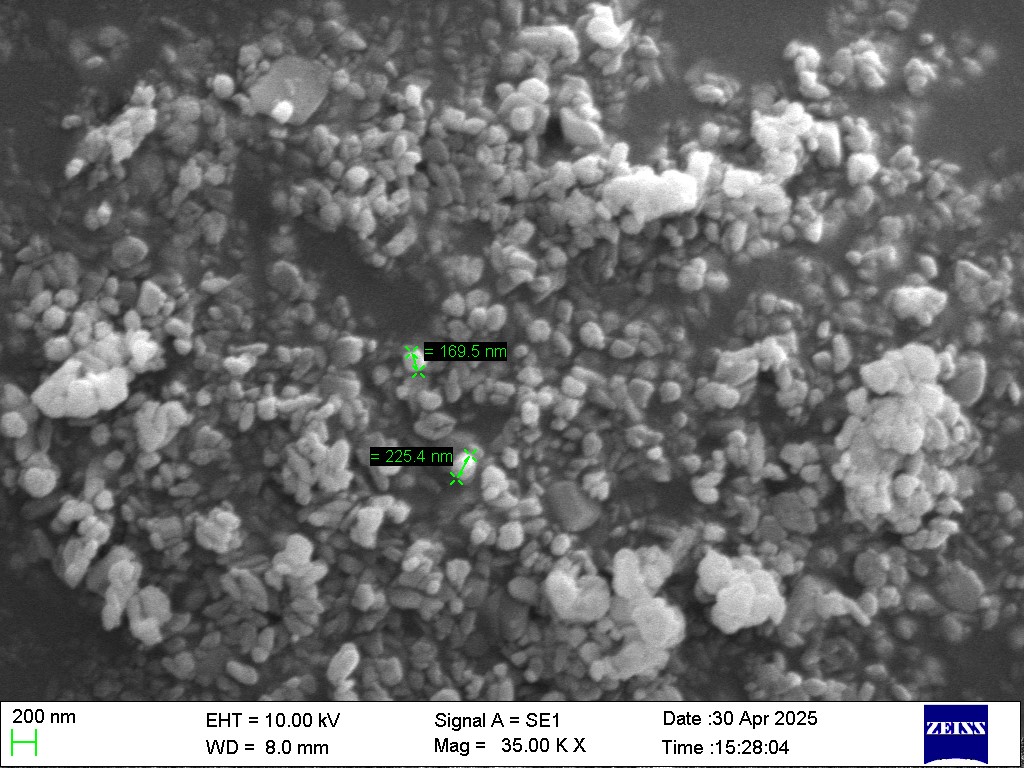


**Figure S11. SEM image of the AgMOF nanoparticulate carrier system showing surface morphology at 35.00 K magnification**

**Tables**

**Table S1.** **Summary of Critical Analytical Attributes (Responses) and Critical Method Parameters (Factors).**

| **Critical Analytical Attributes (CAA) – Responses (Dependent Variables)** | | **Critical Method Parameters (CMP) – Factors (Independent Variables)** | | | | |
| --- | --- | --- | --- | --- | --- | --- |
| **Responses (CAA)** | **Code of CAA** | **Factors (CMP)** | **Code of CMP** | **Units** | **Levels** | |
| Peak Area | R1 |  |  |  | **Low (-1)** | **High (+1)** |
| Retention Time | R2 | Organic Phase (ACN) | A | % | 45.00 | 50.00 |
| Theoretical Plates | R3 | Column Oven Temperature | B | ℃ | 25 | 35 |
| Peak Tailing Factor | R4 | Flow Rate | C | mL/min | 0.8 | 1.0 |

**Table S2. Parameters and results of predicted runs carried out experimentally before screening one optimal run for further validation and assessment based on ANOVA analysis using the Box-Behnken design of Experiment.**

| **Run** | **Mobile Phase (Fig.5)** | **Column oven Temperature** | **Flow Rate** | **Conc.** | **Retention Time** | **Area** | **Height** | **NTP (USP)** | **HETP (USP)** | **Tailing Factor** |
| --- | --- | --- | --- | --- | --- | --- | --- | --- | --- | --- |
| 1. | 47.4% ACN + 52.6% Buffer pH 3 | 27℃ | 0.93 | 5 μg/mL | 3.872 | 544902 | 82297 | 7271 | 20.629 | 1.122 |
| 2. | 46.7% ACN+ 53.3% Buffer pH 3 | 25℃ | 0.80 | 5 μg/mL | 4.609 | 633220 | 85564 | 8242 | 18.199 | 1.154 |
| 3. | 45.2% ACN + 54.8% Buffer pH 3 | 30℃ | 0.99 | 5 μg/mL | 3.782 | 517129 | 81828 | 7285 | 20.591 | 1.161 |
| 4. | 47.5% ACN + 52.5% Buffer pH 3 | 25℃ | 1.00 | 5 μg/mL | 3.626 | 507759 | 80623 | 6925 | 21.662 | 1.126 |
| 5. | 47.5% ACN + 52.5% Buffer pH 3 | 25℃ | 0.80 | 5 μg/mL | 4.521 | 625248 | 83897 | 8032 | 18.676 | 1.127 |
| 6. | 50% Methanol + 50% Buffer pH 3 | 30℃ | 0.80 | 5 μg/mL | 4.267 | 633649 | 48822 | 2849 | 52.644 | 0.748 |
| **7.** | **45% ACN + 55% Buffer pH 3** | **25℃** | **0.95** | **5 μg/mL** | **4.042** | **538865** | **81623** | **7710** | **19.454** | **1.171** |

**Table S3. Validation data representation of the optimized RP-HPLC method (Run 7).**

| **Parameters** | **Description** |
| --- | --- |
| Column | Phenomenex HyperClone BDS C18 130 Å |
| Column size | 4.6 mm × 250 mm × 5µ |
| Column Temperature | 25℃ |
| Mobile phase | Acetonitrile: Acidified water (0.1% v/v Triethylamine, pH 3.0 adjusted with Orthophosphoric acid) (45:55%) |
| Flow rate | 0.95 ml/min |
| Detector and wavelength | UV detector, 371 nm |
| Injection loop capacity | 20 µl |
| Concentration of Samples | 1 ppm or 1 µg/mL (standard and isolated fraction of quercetin) |
| Retention time | 4 min |
| Run time | 12 min |

**Table S4.** **Results of Validation Parameters.**

| **Validation Parameters** | | **Response Parameters** | **Acceptable limit** | | **Observations** |
| --- | --- | --- | --- | --- | --- |
| **System Suitability**  **(1 μg/mL, n=6)** | | Peak Area | RSD < 2.0% | | 80571.17 ± 76.771; RSD = 0.0127 |
|  |  | Retention Time (Rt) | RSD < 2.0% | | 4.068 ± 0.001 mins; RSD = 0.0953 |
|  |  | Theoretical Plates (NTP) | > 2000 | | 7623.167 ± 12.073; RSD = 0.158 |
|  |  | Tailing Factor (Tf) | < 2.0% | | 1.192 ± 0.002 %; RSD = 0.138 |
| **Linear regression data (calibration curve, n=6)** | | Linearity (ng/mL) | | | 50 to 50000 |
|  |  | Regression Equation | | | y = 86.087x+10568 |
|  |  | R^2^ value (> 0.999) | | | 0.9996 |
|  |  | Avg. Peak area Standard deviation (σ) | | | 1050.559 |
|  |  | Limit of Detection (LOD) = [3.3*(σ/s)] | | | 40.271 ng/mL (0.040 μg/mL) |
|  |  | Limit of Quantitation (LOQ) = [10*(σ/s)] | | | 122.035 ng/mL (0.122 μg/mL) |
| **Robustness** | | | | | |
| **Parameters** | | **Average % RSD (acceptable limit < 2.0%)** | | | |
|  |  | **Retention time (mins)** | | **Analyte Peak Area** | |
| Wavelength (±2 nm) | | 0.050 | | 0.311 | |
| pH of the buffer (±0.2) | | 0.207 | | 0.120 | |
| Injection volume (±2 µL) | | 0.052 | | 0.290 | |
| Flow rate (±0.1 mL/min) | | 0.030 | | 0.254 | |
| Mobile Phase ratio (±2% v/v) | | 0.044 | | 0.382 | |
| Column oven temperature (±2 °C) | | 0.031 | | 0.404 | |
| **Precision** | | | | | |
| **Initial conc. (ng/mL)** | | **Average Peak Area (n=6)** | **Standard Deviation** | | **% RSD (Acceptable limit < 2.0%)** |
| 750 ng/mL (Low Quality Control Level) | Repeatability | 65198.500 | 439.529 | | 0.674 |
|  | Inter day | 66260.500 | 824.249 | | 1.244 |
| 1250 ng/mL (Medium Quality Control Level) | Repeatability | 101563.833 | 164.496 | | 0.162 |
|  | Inter day | 101963.833 | 493.213 | | 0.484 |
| 2500 ng/mL (High Quality Control Level) | Repeatability | 216124.083 | 934.865 | | 0.433 |
|  | Inter day | 216641.833 | 1581.173 | | 0.730 |
| **Accuracy (% Recovery)** | | | | | |
| **Initial conc. (ng/mL)** | **% RSD** | **Average Peak Area (n=3)** | **Observed conc. (ng/mL)** | | **% Mean Recovery (98 to 102%)** |
| 750 (75% LQC) | 0.05 | 75145.33 ± 38.812 | 750.140 | | 100.02 |
| 1250 (100% MQC) | 0.82 | 119161.3 ± 979.350 | 1261.437 | | 100.91 |
| 2500 (125% HQC) | 0.87 | 231314 ± 2012.930 | 2564.220 | | 102.50 |
| **Validation Parameter** | | **Response Parameters** | **Freshly prepared samples** | | **Bench-top samples (24 hours)** |
| **Bench-top Stability (24 hours)** | | Average Peak Area (n=4) | 109900.25 ± 846.561 | | 110072.25 ± 619.915 |
|  |  | % RSD (< 2.0%) | 0.77 | | 0.288 |
|  |  | Similarity Index (Close to 1) | 1.0003 | | |

**Table S5.** **Robustness of the developed analytical method.**

| **Parameter (% RSD acceptable limit < 2.0%)** | | **QCT Retention time (mins)** | | **QCT Peak Area** | |
| --- | --- | --- | --- | --- | --- |
|  |  | **Average ± SD (n=3)** | **% RSD** | **Average ± SD (n=3)** | **% RSD** |
| **Wavelength (±2 nm)** | **373 nm** | 4.04 ± 0.002 | 0.051 | 87211.33 ± 279.743 | 0.321 |
|  | **369 nm** | 4.044 ± 0.002 | 0.049 | 87739 ± 264.108 | 0.301 |
| **pH of the buffer (±0.2)** | **2.8** | 4.133 ± 0.008 | 0.206 | 81281.67 ± 173.552 | 0.214 |
|  | **3.2** | 4.127 ± 0.008 | 0.207 | 80887.67 ± 21.595 | 0.027 |
| **Injection volume (±2 µL)** | **18 µL** | 4.0323 ± 954 | 0.014 | 78704 ± 319.235 | 0.406 |
|  | **22 µL** | 4.042 ± 0.004 | 0.089 | 95357 ± 166.334 | 0.174 |
| **Flow rate (±0.1 mL/min)** | **0.85 mL/min** | 4.513 ± 0.001 | 0.034 | 97017 ± 156.694 | 0.161 |
|  | **1.05 mL/min** | 3.658 ± 0.001 | 0.027 | 79511.67 ± 275.555 | 0.347 |
| **Mobile Phase ratio (±2% v/v)** | **43A 57B** | 4.253 ± 0.002 | 0.049 | 85759 ± 351.401 | 0.409 |
|  | **47A 53B** | 3.852 ± 0.001 | 0.040 | 84902.33 ± 300.681 | 0.354 |
| **Column oven temperature (±2 °C)** | **23℃** | 4.082 ± 0.002 | 0.049 | 87773.67 ± 490.164 | 0.558 |
|  | **27℃** | 4.001 ± 0.001 | 0.014 | 88312.33 ± 220.820 | 0.250 |

**Table S6. The table represents the concentration of free drug present in the QCT-loaded AgMOF system, analysed in triplicate to obtain the % RSD values and the final concentration of quercetin detected in 2 mL of 24-hour QCT-AgMOF.**

| **Sr. No** | **Sample ID (n=3)** | **Peak area** | | | **Average** | **Std. Dev.** | **% RSD** | **Back calculated conc. from the equation (ng/mL)** |
| --- | --- | --- | --- | --- | --- | --- | --- | --- |
|  |  | **Trial 1** | **Trial 2** | **Trial 3** |  |  |  |  |
| 1 | A3SN | 15400958 | 15596067 | 15624315 | 15540446.7 | 121623.614 | 0.78 | 180397.489 |
| 2 | A3W1 | 17778827 | 17707327 | 17687769 | 17724641.0 | 47934.547 | 0.27 | 205769.431 |
| 3 | A3W2 | 313963 | 311045 | 312356 | 312454.667 | 1461.500 | 0.47 | 3506.763 |
| 4 | A3W3 | 13969 | 13825 | 13932 | 13908.667 | 74.782 | 0.54 | 38.806 |
| **Total Free drug or unentrapped drug in the 2 mL QCT-AgMOFs dispersion** | | | | | | | | **389712.489 ng/mL** |
